# Fine-mapping of retinal vascular complexity loci identifies Notch regulation as a shared mechanism with myocardial infarction outcomes

**DOI:** 10.1038/s42003-023-04836-9

**Published:** 2023-05-15

**Authors:** Ana Villaplana-Velasco, Marie Pigeyre, Justin Engelmann, Konrad Rawlik, Oriol Canela-Xandri, Claire Tochel, Frida Lona-Durazo, Muthu Rama Krishnan Mookiah, Alex Doney, Esteban J. Parra, Emanuele Trucco, Tom MacGillivray, Kristiina Rannikmae, Albert Tenesa, Erola Pairo-Castineira, Miguel O. Bernabeu

**Affiliations:** 1 The Roslin Institute, Royal (Dick) School of Veterinary Studies, The University of Edinburgh, Edinburgh, Scotland UK; 2 Centre for Medical Informatics, Usher Institute, The University of Edinburgh, Edinburgh, Scotland UK; 3 Population Health Research Institute (PHRI), Department of Medicine, Faculty of Health Sciences, McMaster University, McMaster University, Hamilton, Ontario Canada; 4 MRC Human Genetics Unit, IGC, The University of Edinburgh, Edinburgh, Scotland UK; 5 University of Toronto at Mississauga, Mississauga, Ontario Canada; 6 VAMPIRE project, Computing, School of Science and Engineering, University of Dundee, Dundee, Scotland UK; 7 VAMPIRE project, Centre for Clinical Brain Sciences, The University of Edinburgh, Edinburgh, Scotland UK; 8 The Bayes Centre, The University of Edinburgh, Edinburgh, Scotland UK

**Keywords:** Predictive medicine, Predictive markers, Machine learning, Genome-wide association studies, Myocardial infarction

## Abstract

There is increasing evidence that the complexity of the retinal vasculature measured as fractal dimension, D_f_, might offer earlier insights into the progression of coronary artery disease (CAD) before traditional biomarkers can be detected. This association could be partly explained by a common genetic basis; however, the genetic component of D_f_ is poorly understood. We present a genome-wide association study (GWAS) of 38,000 individuals with white British ancestry from the UK Biobank aimed to comprehensively study the genetic component of D_f_ and analyse its relationship with CAD. We replicated 5 D_f_ loci and found 4 additional loci with suggestive significance (*P* < 1e−05) to contribute to D_f_ variation, which previously were reported in retinal tortuosity and complexity, hypertension, and CAD studies. Significant negative genetic correlation estimates support the inverse relationship between D_f_ and CAD, and between D_f_ and myocardial infarction (MI), one of CAD’s fatal outcomes. Fine-mapping of D_f_ loci revealed Notch signalling regulatory variants supporting a shared mechanism with MI outcomes. We developed a predictive model for MI incident cases, recorded over a 10-year period following clinical and ophthalmic evaluation, combining clinical information, D_f_, and a CAD polygenic risk score. Internal cross-validation demonstrated a considerable improvement in the area under the curve (AUC) of our predictive model (AUC = 0.770 ± 0.001) when comparing with an established risk model, SCORE, (AUC = 0.741 ± 0.002) and extensions thereof leveraging the PRS (AUC = 0.728 ± 0.001). This evidences that D_f_ provides risk information beyond demographic, lifestyle, and genetic risk factors. Our findings shed new light on the genetic basis of D_f_, unveiling a common control with MI, and highlighting the benefits of its application in individualised MI risk prediction.

## Abstract (web summary)

A genome-wide association study finds an inverse relationship between progression of coronary artery disease and retinal vasculature’s complexity measured as fractal dimension, and validates a predictive model for myocardial infarction.

## Introduction

Coronary artery disease (CAD) remains the leading cause of death and disability worldwide^1^. Early diagnosis and preventive therapies are essential strategies to control CAD morbidity and the mortality associated with its outcomes, such as myocardial infarction (MI). There is increasing evidence that morphological changes in the retinal vasculature, for instance in vessel width and vascular complexity, might offer insights into CAD before traditional risk factors (such as systolic blood pressure and cholesterol levels)^2,3^. Recent studies reported that a reduced degree of vascular complexity, quantified through estimates of the fractal dimension (D_f_), is found in individuals who had a higher CAD risk, independent of their age^4^. In one of the most extensive studies to date, Zekavat et al. showed associations between D_f_ and incident CAD, amongst several other conditions^5^. This suggests that D_f_ could be a promising non-invasive and highly accessible biomarker. However, these findings have not translated so far to a substantial increase in prediction accuracy for major adverse cardiac events (MACE) risk when leveraging retinal vascular information in epidemiological models, compared to models based on patient demographics and lifestyle risk factors^6,7^. Likewise, in a landmark study, Poplin et al. developed a deep learning approach capable of accurately predicting some known MACE risk factors from retinal fundus images that only attained marginal improvements in MACE risk estimation compared to known risk factors alone^8^. More recently, Diaz-Pinto et al.^9^ demonstrated another deep-learning-based model capable of predicting two measures of left ventricular mass volume, recognised as MI biomarkers, from fundus images and subsequently showed risk prediction improvement over a demographic-based risk model (including age, sex, SBP, DBP, cholesterol levels, glucose levels, Hba1c, daily alcohol intake and smoking status). However, it remains unknown whether these ‘*blackbox’* approaches leverage vascular information or otherwise. Finally, little is known about the degree of overlap between MI risk information provided by D_f_ and established genetic risk factors. Such knowledge would provide invaluable data for untangling genetic and environmental contributors. Beyond retinal vascular structural phenotyping, Theuerle et al. showed the potential of functional testing of retinal microvasculature for the prediction of MACE risk^10^. However, it remains unclear what improvement functional testing offers over the ubiquity of retinal fundus photography.

Evidence points towards coronary and retinal vessels experiencing similar pathophysiological changes at even early CAD stages^11–14^, plausibly influenced by a shared genetic basis^13,15–20^. Population-based studies demonstrated that both tortuosity and width of arteries and veins have a genetic basis^16,17^. Veluchamy et al. described two novel loci near the *COL4A2* and *ACTN4* genes associated with retinal tortuosity, previously reported in genetic atrial fibrillation and CAD^17^ studies. During the preparation of this manuscript, a genome-wide association study (GWAS) was published identifying 7 loci contributing to D_f_^5^. Zekavat et al.^5^ calculated D_f_ from available fundus images of a subset of 54,813 multi-ancestry participants in the UK Biobank cohort. That study, however, did not investigate shared D_f_ and MI molecular regulation and the GWAS is based on a linear model with multiple ancestries that do not account for individuals’ genomic relatedness.

**Fig. 1:**
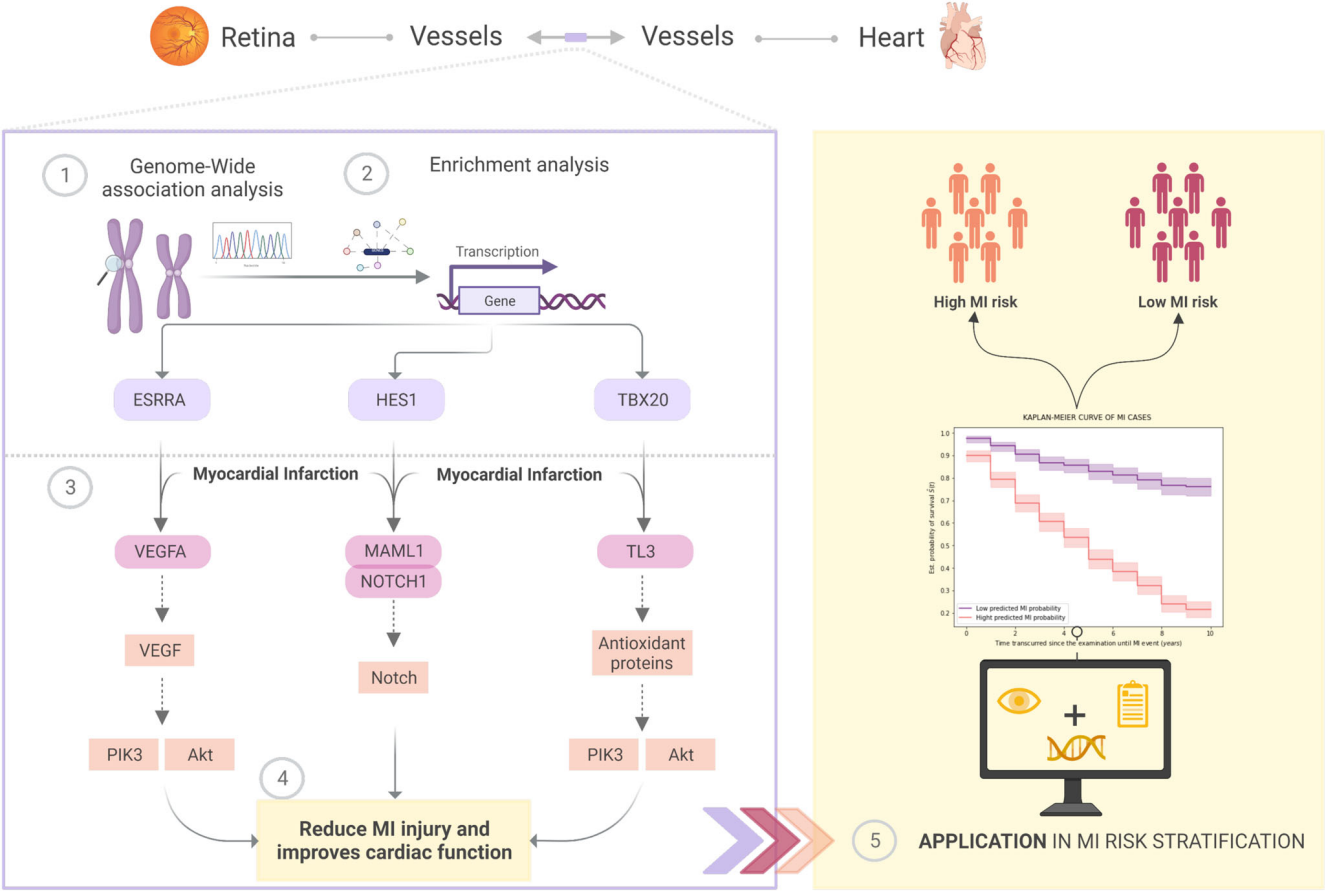
Study results and application to stratify MI risk in UKBB. The authors created this figure with BioRender.com.

We report here a GWAS of D_f_, from ~38,000 white-British participants from the UK Biobank. The aim is twofold: to comprehensively study the genetic control of D_f_ and to assess the extent of its relationship with CAD (Fig. 1). We replicated 5 D_f_ loci and found 4 additional loci that are suggestive to contribute to D_f_ variation. Two of these loci (*SLC12A9* and *RDH5* genes) were previously associated with cardiovascular risk factors and diseases^21^. Genetic correlation estimates indicate a shared genetic signal between D_f_ and CAD, suggesting that decreasing D_f_ might be influenced by clinical CAD manifestations and, in part, by common genetic effects. Fine-mapping and enrichment analysis on D_f_ loci identified Notch signalling regulatory variants supporting a shared mechanism with MI outcomes. Given this strong connection, we developed a model to predict incident MI cases in the UK Biobank over the 10 years following ophthalmic examination at baseline, including D_f_ and a CAD polygenic risk score (PRS_CAD_). Internal 10-fold cross-validation shows a considerable performance improvement compared with the SCORE model^22^, an established CAD risk prediction score based on epidemiological variables. This enhancement can be partly explained by the additional predictive power of retinal and genetic determinants, as these respectively capture early vascular morphological abnormalities and personalised MI risk (Fig. 1). Furthermore, our ablation study demonstrates that our model improves on an extension of SCORE including PRS_CAD_, evidencing that Df provides risk information beyond epidemiological and genetic risk factors in a population subset of UKBB. Our findings shed new light on the genetic component of D_f_, suggesting an intricate common genetic basis with CAD aetiology, and demonstrate its potential for individual MI risk prediction.

## Results

### Automated quality control and fractal dimension calculation in UK Biobank fundus images reveal interocular asymmetry in vascular complexity at an individual level

**Fig. 2:**
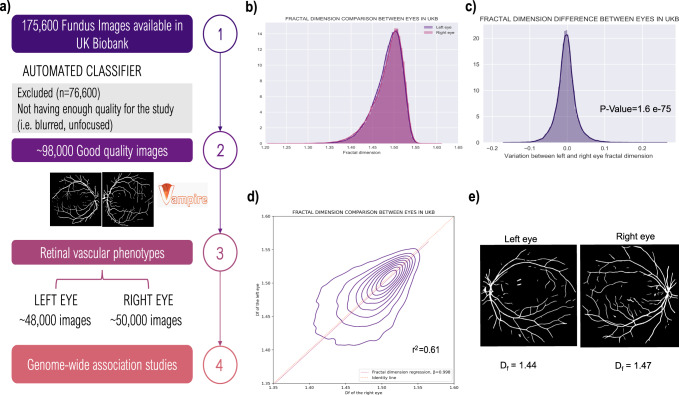
Pipeline and D_f_ characteristics. **a** Study design diagram describing the stepwise development of this project. **b** Left and right D_f_ histogram. **c** Individual variation distribution between left and right D_f_. **d** Overlapping left and right D_f_ histograms including the regression line. **e** Example of individual interocular asymmetry in UKBB fundus images.

For this study, we first developed a semi-automated pipeline to segment the vasculature and select good-quality segmentations in 175,611 fundus images available in the UK Biobank (Fig. 2a) using VAMPIRE software (version 3.1, Universities of Edinburgh, and Dundee)^23,24^, and a previously published fundus image classifier^25^. An image quality score (IQS) was computed as part of the classification process (see section “Methods”). D_f_ was subsequently calculated from binary vessel maps produced automatically by VAMPIRE for ~98,600 good-quality images.

We completed the, to our knowledge, largest within individual interocular D_f_ comparison (*n* = 39,656 participants) reported so far. The population median (1.492 ± 0.043) and D_f_ distributions appear identical between left and right eyes (Fig. 2b and Supplementary Data 1). However, their moderate correlation (r = 0.61, *P*-value = 2 × 10^−16^ Fig. 2d) and the significant difference between left and right D_f_ (paired T-test *P*-value = 1.59 × 10^−75^) highlight an individual interocular asymmetry (Fig. 2c), where 50% of the individuals have a right D_f_ 1 SD unit larger than their respective left D_f_. As shown in Fig. 2d, differences occur in both directions and are more pronounced when any of the D_f_ is lower than the median. To control for this individual asymmetrical effect (Fig. 2e), we performed further analysis in both eyes separately.

We next fitted univariate linear models using D_f_ as the dependent variable and estimated the Pearson correlation between D_f_ and 779 UKBB binary and quantitative traits (see “Methods”) and IQS. Amongst these 780 variables, IQS has the strongest effect (β_right_ = 0.033, *P*-value < 10^−300^; β_left_ = 0.024, *P*-value < 10^−300^; r^2^_right_ = 0.39, *P*-value < 10^−300^; r^2^_left_ = 0.36, *P*-value < 10^−300^). Supplementary Figure 1 illustrates this association and that a larger interocular IQS difference moderately affects D_f_ variation (β = 0.014, *P*-value < 10^−300^). Therefore, we account for IQS influence in our following analysis.

Besides IQS, 75 quantitative and 161 binary traits were significantly associated with D_f_ after Bonferroni correction^26^ (*P*-value < 0.05/780 = 6.41 × 10^−5^). Age, sex, height, retinal disorders, smoking, hypertension, and CAD have the greatest significant effect on D_f_ in both eyes amongst all measurements (Supplementary Data 2).

### Fine-mapping reveals nine fractal dimension loci and their association with cardiovascular risk factors

**Fig. 3:**
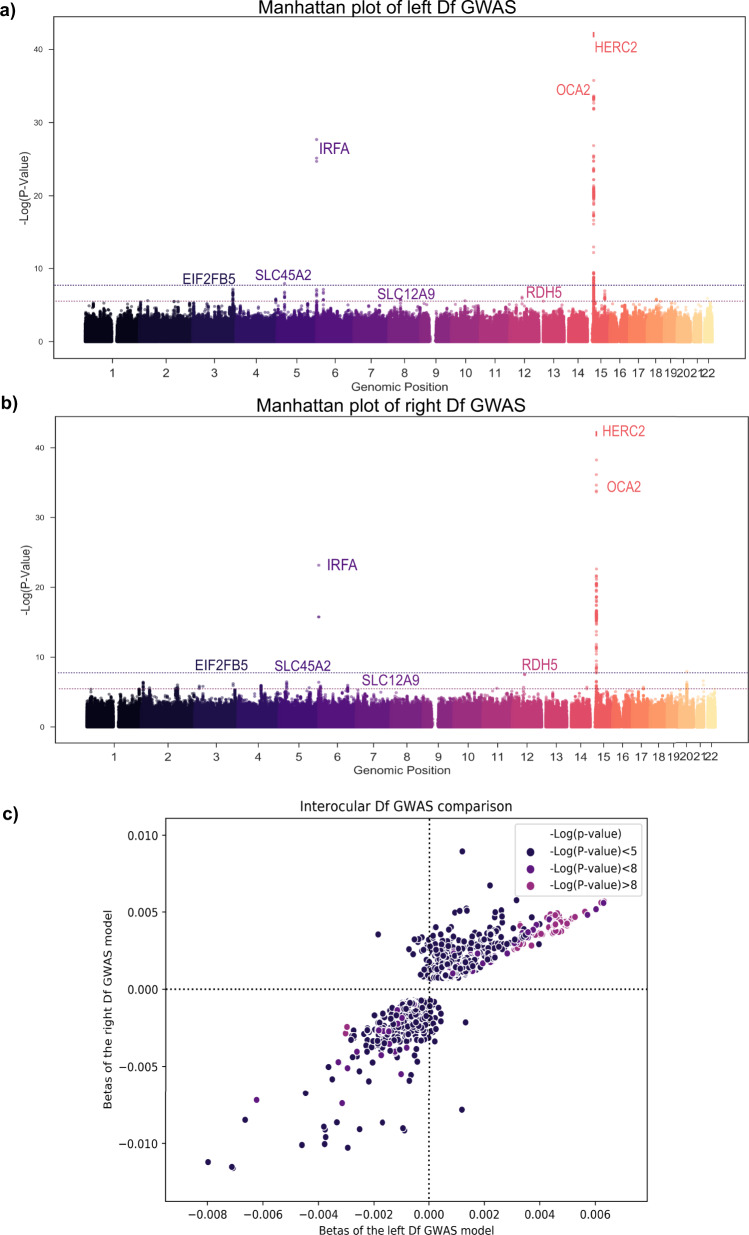
GWAS of both eyes’ D_f_. Manhattan plot of **a** left (top) and **b** right (bottom) D_f_. Points are truncated at –log10(P) = 50 for clarity. **c** Comparison of the genetic variant effects between left and right D_f_ results. Colour depth indicates the significance of each variant (navy, violet, and purple for non-significant, close to genome-wide significance and significant, respectively). Genetic variants included are truncated at a minimum –log10(p) = 3 for clarity.

Here we present a GWAS on D_f_. This was completed with 38,811 and 38,017 unrelated white-British UK Biobank participants that had a right and left D_f_ measure, respectively. After QC (see “Methods”), there were 9,275,849 imputed SNPs with HWE > 10^−6^, MAF > 5 × 10^−3^, a call rate >0.9, and an imputation score >0.9. The GWAS model included hair and skin colour to control for spurious associations given the influence of eye and skin colour on fundus colour^27,28^. Hair colour replaced eye colour because the latter is not recorded during UKBB assessments. In addition, we completed a supplementary GWAS including an eye colour PRS based on the study by Lona-Durazo et al.^29^, which indicated no eye colour effect in our GWAS results (see section “Methods”). The quantile-quantile plot of both GWASs indicated an adequate control of the genomic inflation in our analysis (λ_GC_ = 1.065 and λ_GC_= 1.067 in the right and left eye, respectively, see Supplementary Figure 2). Fig. 3c illustrates the SNPs effects comparison between eyes GWAS studies, highlighting analogous results. Furthermore, an additional GWAS of mean D_f_ including participants from both left and right eye populations (see “Methods” section) reported equivalent SNP associations to those from eye-specific populations (Supplementary Figure 3). The genetic correlation estimates close to 1 between mean D_f_ and eye-specific GWAS (Mean D_f_ and right D_f_: 0.93 ± 0.03, *P*-value = 3.89e−201; mean D_f_ and left D_f_: 0.89 ± 0.07, *P*-value = 2.62e−38) revealed that mean D_f_ GWAS was equivalent to those of left and right D_f_ measures.

Fine-mapping analysis of D_f_ GWAS observations indicated that there were nine independent credible SNP sets with a posterior inclusion probability (PIP) > 0.95 (Supplementary Table 1). The credible SNP sets with strongest associations were located at *OCA2* (rs72714116, *P*-value = 7.41 × 10^−48^) and *HERC2* (rs12913832, *P*-value = 2.16 × 10^−96^) genes in chromosome 15 (Fig. 3a, b and Supplementary Table 2). We observed another significant association near *IRF4* gene (rs12203592, *P*-value = 6.59 × 10^−24^). These results are consistent with Zekavat et al. GWAS. Phenome-wide association studies (PheWAS), using GeneATLAS^30^ and GWASCatalog^31^, have commonly reported these SNPs in skin, hair, and eye colour analyses. Recent ocular studies demonstrated their implication in lens disorders, cataract, glaucoma, visual acuity, and retinal venular and arteriolar width and tortuosity^5,17,20,28,32–40^.

In addition to these regions, we found 4 credible SNPs that had suggestive significance (*P*-value < 10^−06^) which did not reach genomic-wide significance (Table 1). The SNP located at *SLC45A2* gene was previously reported in pigmentation analyses^29,41^, whereas those near *EIF2B5* and *AGPAT3* genes were described in blood content and inflammation GWASs^34,36,42^. Those SNPs located at *RDH5/ORMLD2* and *AGPAT3* genes also have a strong effect on multiple ocular traits and diseases (such as macular thickness and retinal detachment), hypertension, and arterial disorders. The effect of the SNP at *SLC45A2* gene is in line with Zekavat et al.^5^ results. We could not make a complete comparison between studies as the available summary statistics are truncated at a *P*-value = 10^−4^. The comparison between reported variants is in Supplementary Table 3.

**Table 1:** Summary statistics of summary statistics of D_f_-associated SNPs and its nearest located gene.

	Right Df			Left Df			Nearest gene
SNP	BETA	SD	−Log (*P*-value)	BETA	SD	−Log (*P*-value)	
rs73175105	−1.83E−03	3.33E−04	5.33	−1.01E−04	3.60E−04	5.22	*EIF2B5*
rs16891982	3.53E−03	6.94E−04	6.46	3.75E−03	6.59E−04	7.93	*SLC45A2*
rs12203592	−2.85E−03	2.80E−04	23.62	−2.31E−03	2.68E−04	28.67	*IRF4*
rs6018400	−1.06E−03	2.48E−04	5.72	−1.07E−03	2.37E−04	5.17	*RDH5/ORMLD2*
rs12913832	5.65E−03	2.71E−04	96.97	6.34E−03	2.58E−04	131.28	*HERC2*
rs72714116	4.20E−03	6.35E−04	51.76	3.33E−03	5.99E−04	27.07	*OCA2*
rs73226964	−4.00E−03	7.75E−04	6.63	−4.21E−03	7.48E−04	4.63	*AGPAT3*

The SNP heritability (h^2^_SNP_) of the left and right D_f_ estimate are, respectively, 0.09 ± 0.015 and 0.10 ± 0.014. These h^2^_SNP_ magnitude is in line with previous results from retinal vascular tortuosity^17,20^, retinal width^18^, and the recently published D_f_^5^ GWAS.

We completed additional D_f_ GWAS using independent UKBB participants with European (n_left_ = 4340 and n_right_ = 4288), Asian (n_left_ = 562 and n_right_ = 568), and African (n_left_ = 498 and n_right_ = 509) ancestry to assess if these populations replicated our observations. Only the GWAS including participants with a white European ancestry replicated the strongest associations (*P*-value < 0.05/9 = 0.0056), which can be explained by the considerably larger number of participants in this analysis when compared with Asian and African ancestries. Little heterogeneity and forest plots of D_f_ loci indicate that multiple significant genetic variants (rs16891982, rs12203592, rs12913832 and rs31381412) have a similar effect across Asian, African, European, and white-British ancestries (Supplementary Fig 4).

We complemented the replication of our GWAS results with an association study in the Canadian Longitudinal Study on Aging (CLSA). This consisted on fitting a linear regression on D_f_ that controlled for the 20 first principal components and a genetic risk score (GRS) for D_f_, which was estimated using the summary statistics of the GWAS reported above (see “Methods”). We found that the D_f_ GRS had a significant effect on left, right and mean D_f_ phenotypes (Table 2), suggesting thus that the SNPs previously described in the UKBB GWAS contribute to D_f_ variation in the CLSA population.

**Table 2:** Association estimates between D_f_ measures and its respective GRS in the CLSA population.

CLSA models	Estimate	SE	*P*-value
Df (both eyes)* (*n* = 16,205)	0.0212	0.0024	<2E16
Df (right eye) (*n* = 14,820)	0.0225	0.0026	<2E−16
Df (left eye) (*n* = 11,826)	0.0220	0.0030	5.33E−13

*measure from the best quality image of either sides, or average of both sides when both images were of similar quality.

### Genetic correlation estimates and functional analysis indicate shared genetic signal between fractal dimension and coronary artery disease

To assess the link between D_f_ and CAD risk factors and outcomes, we calculated their genome-wide genetic correlation using LD score regression (LDSC)^43^. Genetic correlation estimates (r_g_) indicated a negative correlation between D_f_ and hypertension (r_g _= −0.30, *P*-value = 4.52 × 10^−06^), acute MI (r_g_ = −0.16, *P*-value = 0.03), and CAD (r_g_ = −0.18, *P*-value = 0.025) (Table 3). All these estimates agree in direction with phenotypic correlations (see Supplementary Data 2) and published studies, which reported that retinal D_f_ decreases as people develop these conditions^2,3,11,44^. Therefore, our results suggest that these correlations of phenotypes could be partly explained by its shared genetic basis.

**Table 3:** Genetic correlation estimates and significance (*P*-value) between D_f_ and associated cardiovascular events.

Correlated trait	LEFT EYE			RIGHT EYE		
	${{{\mbox{r}}}}_{{{\mbox{g}}}}$	SE	*P* value	${{{\mbox{r}}}}_{{{\mbox{g}}}}$	SE	*P* value
Hypertension	−0.2229	0.0534	3.026E−05	−0.3020	0.0659	4.52E−06
Acute myocardial infarction	−0.1717	0.0809	0.0801	−0.1585	0.1075	0.0308
Self-reported acute myocardial infarction	−0.2071	0.0754	0.006	−0.2663	0.0982	0.0067
Coronary artery disease	−0.2214	0.0591	1.785E−04	−0.1776	0.0795	0.025
Atherosclerosis	−0.3585	0.1819	0.084	−0.2668	0.2100	0.051
Right Fractal dimension	0.9468	0.0962	1.75E−26	–	–	–

Moreover, we estimated the r_g_ between pigmentation traits and D_f_ to examine the similarities in their genetic basis (Supplementary Fig 5). Although the estimates are non-significant (r_g_ = −0.0751, *P*-value = 0.64), local genetic correlation near to GWAS peaks may be significant.

We investigated possible causal relationships between CAD, hypertension, MI and D_f_ using Mendelian randomisation. We found evidence of horizontal pleiotropy on the loci of interest (pleiotropy analysis *P*-value = 0.0056), which indicated that we are unable to infer the causality between D_f_ and such cardiovascular events (Supplementary Table 4).

### A subset of credible genetic variants points towards associated myocardial infarction post-conditioning signalling pathways

We examined the potential for transcription factor binding site (TFBS) disruption of the lead snps from each credible set from the fine-mapping analysis. We observed 20 TFBS with a strong disruptive effect described in Supplementary Table 5. Eight of these TFBS remained significant after applying a more restrictive threshold to the predicted disruptiveness of its activity between reference and alternative alleles (|AlleleDif| > 1.5). We investigated those associated TF whose binding activity influenced the expression of a gene within 150 kb in the chromosome. This left us with 4 D_f_ SNPs, 5 TFBS and 9 regulated genes. Protein–protein interaction networks show that these TFs and regulated genes participate in Notch and VEGF signalling pathways. Numerous studies indicate that the upregulation of both signalling pathways after an MI event leads to reduced infarct size, improved angiogenesis, and cardiac function, increasing the survival rate and limiting cardiac injury^45–47^.

### Fractal dimension improves prediction of incident myocardial infarction in UK Biobank cases

**Fig. 4:**
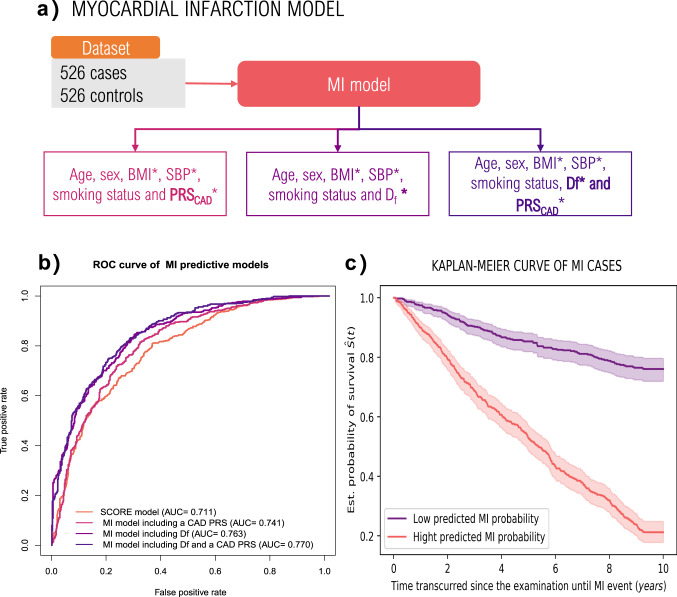
Development and performance of MI predictive models. **a** Diagram illustrating the development of our MI model. **b** ROC curve of MI predictive models. **c** Kaplan–Meier curve of incident MI cases separated by predicted MI probability. * D_f_ fractal dimension, PRS_CAD_ CAD polygenic risk score, BMI body-mass index, SBP systolic blood pressure.

Given our findings, we hypothesized that D_f_ and PRS_CAD_ can provide additional information for MI risk estimation at an individual patient level. We thus developed a model to predict incident cases of MI over the 10 years following ophthalmic examination at baseline (Fig. 4a). Briefly, the model includes PRS_CAD_ derived from a meta-analysis completed by the CARDIoGRAMplusC4D Consortium^21^, clinical variables from an established CAD risk assessment strategy named SCORE^22^ (age, sex, smoking status, SBP and BMI), and the D_f_ of both eyes. We also considered model versions excluding either PRS_CAD_ or D_f_ to elucidate their independent effect. As a baseline for comparison, we retrained the original SCORE model^22^. The MI model was trained with the 526 individuals who experienced an MI event after their UKBB ophthalmic examination. We created a control group with an equal number of individuals with an equivalent age range and had no underlying MI and CAD (Supplementary Table 6). The mean age and SD in the case and control group are respectively 57.31 ± 6.47 and 54.21 ± 7.84 years. We chose the random forest classifier (RFC) as this method allows one to model non-linear associations with the outcome and interactions between the predictor variables, which boosts the prediction while being interpretable^48,49^. Internal 10-fold cross-validation (FCV) indicates that our models dominate the ROC curve of the SCORE model, achieving a greater precision, recall, and AUC (Fig. 4b and Table 4). Amongst our considered models, the model including PRS_CAD_ (AUC = 0.741 ± 0.001) yielded an AUC significantly different from the one introducing D_f_ (AUC = 0.763 ± 0.001), and the one combining both D_f_ and PRS_CAD_ (AUC = 0.770 ± 0.001) (Table 4 and Supplementary Table 7). Additional assessments in our proposed model indicated that the replacement of D_f_ measures with D_f_ adjusted by IQS, the introduction of one-eye D_f_ measurements in our MI model, or the use of mean D_f_ in our model yielded a comparable performance to the aforementioned ones (Supplementary Table 8).

**Table 4:** Internal 10-fold cross-validation of MI models evaluated with precision, recall and AUC.

	MI			
Model	Precision (95%CI)	Sensitivity (95%CI)	Specificity (95%CI)	AUC (95%CI)
SCORE model^22^	0.716 (0.664–0.741)	0.725 (0.691–0.767)	0.691 (0.652–0.731)	0.719 (0.681–0.737)
Random Forest including PRS_CAD_	0.735 (0.708–0.782)	0.756 (0.726–0.801)	0.739 (0.702–0.777)	0.741 (0.725–0.775)^a^
Random Forest including both eye-specific Df	0.756 (0.732– 0.802)	0.778 (0.762–0.831)	0.758 (0.721–0.795)	0.763 (0.750–0.802)^a^
Random Forest including mean Df and PRS_CAD_	0.733 (0.716–0.770)	0.779 (0.743–0.814)	0.756 (0.717–0.797)	0.748 (0.722– 0.773)^a^
Random Forest including Df and PRS_CAD_	0.770 (0.734–0.805)	0.790 (0.757–0.826)	0.764 (0.728–0.800)	0.770 (0.751–0.802)^a^

^a^AUC estimates significantly different (Wilcoxson signed-rank test *P*-value < 0.005) from the ones obtained with the SCORE model. The obtained Wilcoxon signed-rank *P*-value for each model comparison is included in Supplementary Table 7.

Next, we investigated survival rate differences between low and high MI-risk groups. These groups were defined by the predictions obtained with our top-performing MI model and by subsequently separating these with a probability threshold of 0.5 (high MI-risk>0.5 and low MI-risk = <0.5). The Kaplan-Meier curve (Fig. 4c) illustrates a significant divergence between these groups (Log-rank test *P* = 3.52 × 10^−30^), which can be explained by the pronounced decrease in survival during the first 4 years in the high MI risk group.

Finally, we performed an ablation study to understand the origin of the performance improvement in our new model. Briefly, we evaluated the performance of all possible variations between SCORE and the top-performing model (see “Methods” section). This assessment revealed three key contributors to the reported improvement: the use of quantitative variables, the introduction of PRS_CAD_ and D_f_, and the use of a random forest classifier. An extended discussion can be found in Supplementary Table 9. The added predictive value of D_f_ is supported by the RFC development analysis which reveals that age, BMI and D_f_ are the most important features in its architecture (Supplementary Figure 6). PRS_CAD_ is also a determinant of the model’s development as its RFC importance is equivalent to SBP and smoking taken together, which is in line with recently published results^14^.

## Discussion

This work provides a comprehensive examination of the D_f_ genetic basis, unveiling regulatory mechanisms at the Notch signalling pathway that contribute to an intricate shared genetic basis with MI. Given the strong D_f_ and MI connection, we presented a predictive model for MI based on a random forest algorithm that includes D_f_ and a CAD polygenic risk score (Fig. 1). This novel model improves MI individual risk prediction compared to state-of-the-art approaches, demonstrating the additional predictive power of these complimentary traits to early identify high-risk groups.

We identified an individual interocular D_f_ asymmetry in UKBB that led us to perform most of the analyses in both eyes separately. This finding is in line with published studies that reported lateral asymmetry in D_f_, tortuosity, and retinal width^50^. We observed that this asymmetry is more pronounced when one of the two eyes has a D_f_ below the population median. Interestingly, the regression coefficients and the Pearson’s correlation estimates between D_f_ and UKBB traits, and the genetic findings are equivalent in both eyes independently, suggesting that the asymmetrical effect has a negligible influence at a population level. A quantitative assessment of the asymmetry of retinal vascular measurements between eyes seems crucial for studies on retinal vascular biomarkers, often conducted on a single eye, and require further work.

We found that age, sex, smoking, and developing ocular and cardiovascular diseases have a significant effect on D_f_, agreeing with studies reporting that D_f_ decreases with age or by developing these conditions^2,11,15,19^. Interestingly, IQS has the strongest effect on this trait. To overcome quality imaging differences, numerous studies elaborate on the importance of assessing quantitatively image quality, especially in large cohorts analysed automatically^51^. In our case, IQS is computed from the binary vessel map and encapsulates the vessels segmentation’s sharpness and connectivity, which are key features frequently used^51,52^ to compute vascular branching complexity.

We replicated the effect of 5 loci associated with D_f_ with similar effects across European, Asian, and African UKBB participants. We found 4 loci close to genome-wide significance that are suggestive to contribute to D_f_. The effect of these 4 novel loci could not be compared with Zekavat et al.^5^ due to the *P*-value < 10^−04^ truncation in their summary statistics. Nevertheless, differences between both D_f_ GWAS can be attributed to our different strategies as the previously published GWAS combines multiple ancestries and does not control for individuals' relatedness, increasing then the type I error. Furthermore, published tortuosity GWAS^17,20^ reported the significant effect of COL4A2 and ACTN4 genes. Neither this study nor the Zekavat et al.^5^ paper found an association at these genomic regions, suggesting that D_f_ and tortuosity also have distinct associated loci contributing to their regulation, which is consistent with published GWAS in retinal width and tortuosity^17,20,32^.

Most of the genetic variants we report here are relevant to multiple traits and diseases; for instance, the one located near *HERC2* has been previously associated with hair^33^, skin^40^, and eye colour^35^; but recent studies also suggest a strong effect in AMD^36^, glaucoma^37^, intraocular pressure^38^, visual acuity^39^, retinal arterial width^18,32^, retinal vascular complexity and density^5^, and arterial and venular retinal tortuosity^17,20^. Another interesting associated SNP is the one near the *SLC12A9* gene as it has been reported in pigmentation^33,35,40^, mean arterial pressure^34^, and resting heart rate^42^ GWAS. We found significant negative r^2^_g_ estimates between D_f_ and hypertension, CAD, and MI. The direction of these estimates agrees with their phenotypical correlations and published papers^4,5,11^, suggesting the correlation of phenotypes is influenced by its genetic correlation. This finding agrees with four aforementioned studies^5,17,20,32^ which identified novel retinal width and tortuosity loci associated with CAD but did not estimate a genetic correlation between retinal phenotypes and CAD.

**Fig. 5:**
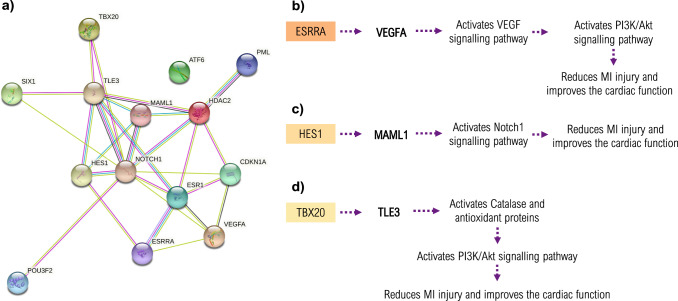
Enrichment analysis of D_f_ loci. **a** Protein–Protein interaction network of enriched TF and regulated genes. Upregulation of **b** *ESRRA* (top), **c** *HES1* (middle) and **d** *TBX20* (bottom) in *VEGF* and Notch signalling pathway after an MI event.

We complemented our functional analysis with in silico TFBS disruptiveness prediction of credible variants. We observed four credible D_f_ gene sets with a strong disruptive effect in 5 TFBS and 9 regulated genes, which participate at different Notch signalling pathway stages (Fig. 5). One possible mechanism to modulate its activity is through the alteration of *ESRRA* binding affinity, which influences *VEGFA* transcription. In-vitro and animal model studies indicate that after an MI event, *VEGFA* upregulation activates *VEGF* signalling pathway, which has a cross-link with Notch pathway and increases its activity^45,53–55^. Another mechanism derives from *HES1* binding site affinity. *HES1* influences *MAML1* and *NOTCH1* expression and directly affect Notch signalling^45–47,56^. The last mechanism influencing Notch activity is mediated through *TBX20* binding site affinity, which plays a role in *TLE3* transcription. Under a MI event, multiple studies indicate that *TLE3* upregulation activates PI3K/Akt signalling pathway, a downstream process of Notch signalling pathway^57,58^. Numerous in-vitro and animal models studies support that this increased Notch activity, mediated by *HES1*, *ESRRA* and *TBX20* upregulation, leads to reduction of cellular oxidative stress consequently improving myocardial viability, regeneration, and survival rate after a MI event^45–47^. We hypothesize that the TF binding disruption caused by these genetic variants influence Notch activity and, in the case of MI, might have a risk-conferring effect^46,47,56–62^. Furthermore, the alleles which predict a stronger TFBS disruptiveness have a negative effect size on D_f_ (see Supplementary Figure 7). Then, we could speculate that individuals with higher D_f_ might not have a disrupted Notch signalling pathway, which might be protective towards the response of a myocardial infarction event. An extended discussion is available in the Supplementary Table 5. Thus, these analyses suggest that there is an intricate shared genetic basis between vascular complexity and MI and further in-vitro experiments are needed to characterise gene expression and regulation of retinal tissue to better understand it.

The potential of the retinal vasculature for stratifying the risk of Major Adverse Cardiac Events (MACE) has already been assessed in diabetic^6,7^ and non-diabetic^8,10^ individuals. Several predictive models have included retinal traits, either in a semantic^6^ or a non-semantic construction^8^, but reported very modest improvements in terms of AUC compared to the established risk estimation strategies based on epidemiological variables (e.g., 0.73 vs 0.72 in ref. ^8^). This discrepancy with our results might be attributed to the different clinical definitions of MACE, comprising normally a heterogeneous group of cardiovascular events where some of which might be not well captured in secondary care data. This situation reduces the model’s statistical power as there might be an overlap in case-control groups. In the case of diabetic population studies, both cases and controls also have comorbidities directly affecting the architecture of the retinal vasculature that might reduce predictive power for MACE risk. Theuerle et al.^10^ reported that retinal arterial dilation response to induced flickering light (FI-RAD) promisingly stratified MACE risk over 200 individuals from a local medical centre. Even though they found CAD family history and reduced FI-RAD to be the strongest MACE risk predictors, no comparison with traditional models is described. We could not assess the effect of this functional phenotype in this work as its computation derives from an invasive procedure that is not possible to apply retrospectively to existing imaging repositories (e.g. UKBB, SCONe). In this work, we focused on retinal structural variations, and MI events, and considered available ICD10 guidelines and UKBB validation reports of MI data to characterize cases, achieving the maximum possible statistical power.

Recent papers have addressed the additional predictive value of a CAD PRS in MACE and CAD risk stratification^6,7,14,63–65^. These approaches, although mainly developed in European populations, achieve a better identification of high-risk MI individuals than those strategies based only on epidemiological variables^14,63–65^. Given this promising finding and the observed shared genetic basis between D_f_ and MI, we examined the effect of both retinal and genetic determinants on MI event risk stratification. We found that adequate clinical phenotyping is key to our models’ performance, but, as shown by our ablation study, the choice of the random forest algorithm, the use of continuous variables and the introduction of D_f_ and PRS_CAD_ in the model all independently improve traditional individual MI risk predictions in this moderate population of study. In addition, the model including these three modifications achieves the greatest performance. D_f_ thus provides an early indication of coronary abnormalities not fully captured in clinical variables of these participants and that PRS accounts for the individual protective/risk-conferring effect on the genetic architecture of the disease. Hence, the proposed model has the potential, as illustrated in the Kaplan-Meier analysis, to stratify UKBB individuals by MI risk. This could be applicable to equivalent populations and after further external validations, allow for early targeted preventive efforts, like the administration of cholesterol-lowering treatments.

Our work has multiple limitations. Firstly, there are only 526 MI cases with a good-quality fundus image taken in UKBB. Higher numbers of such participants would allow us to train and evaluate our models more robustly. Secondly, the study population for the predictive models only consisted of UKBB participants with European ancestry with similar sociodemographic status, restricting the application of translational strategies to non-white European and British individuals among different sociodemographic profiles. Furthermore, the PRS included in the proposed predictive model is based on a meta-analysis completed with participants with mainly white European non-British and white British ancestries. Then, it is of utmost importance to complete GWAS in non-European populations to provide input for PRS estimations so that they are included in such medical applications. Thirdly, the stability of numerical estimates of the fractal dimension is the object of a continuing debate in the retinal image analysis community^66–68^. Fourth, we did not have an external validation cohort to complete an external validation of our MI model. This matter is attributed to the lack of available datasets containing extensive phenotyping from its participants. Finally, there is little information about the genetic expression profiles and the regulation mechanisms of retinal and ocular tissues in public databases. This might be influenced by the minority of studies across these tissues and the complicated protocols to extract and characterise them.

In conclusion, our study contributes to a growing body of evidence showing associations between abnormal morphologic characteristics in coronary vessels and retinal vascular remodelling. In particular, we found that credible fractal dimension loci modulate Notch signalling regulation, and partly explains the intricate shared genetic basis with MI. Remarkably, our MI model improved the stratification of the high-risk population. This is of great interest as it discloses a promising holistic strategy that can prevent MI incidence and triage those with an elevated MI hazard. This study ultimately sheds new light on the value of easily accessible vascular imaging phenotypes and their promising application in personalised medicine.

## Methods

### UK Biobank

UK Biobank (https://www.ukbiobank.ac.uk) is a large multi-site cohort study that consists of 502,655 individuals aged between 40 and 69 years at baseline, recruited from 22 centres across the UK during 2006–2010. The study was approved by the National Research Ethics Committee, reference 11/NW/0382, and informed consent was obtained from all participants as part of the recruitment and assessment process. From these, a baseline questionnaire, physical measurements, and biological samples were undertaken for each participant. Ophthalmic examination was not included in the original baseline assessment and was introduced as an enhancement in 6 UKBB centres across the UK. This examination consisted on capturing paired retinal fundus with a 45º primary field of view obtained with Topcon 3D OCT-1000 MKII (Topcon Corporation). This project was completed using fundus images collected in the first and the repeated ophthalmic examination which took place in 2012 and 2013. It includes 175,709 fundus images (87,552 left and 88,157 from the right) from 67,725 participants.

### Image classification

Image quality was not reported in the UKBB cohort and was found wanting for the purpose of automatic analysis in the first study of this kind^69^. A previous study defined an automated classifier for this dataset using three imaging features following vessels segmentation: white pixel ratio (WPR), largest connected component ratio (LCCR) and the number of connected components (NCC) on a support vector machine (SVM) classifier^25^. We reproduced this classifier using a data subset of 448 random fundus images and VAMPIRE 3.1 software running in MATLAB 2018a^23,24^. The software performs automatic detection of the retinal vasculature, creating a binary vessel map for each image. A.V.V. manually classified the quality of these images based on the connectivity and the sharpness of the binary vessel map, and the lack of imaging artefacts. Manual classification was repeated 2 times using the same random subset of 100 images and the intra-classifier agreement coefficient was 0.897. This dataset was subsequently split in a training (*n* = 278) and validation (*n* = 170) sets. Both data subsets included an even number of manually classified good and bad quality images. We obtained a precision of 0.95, and a recall of 0.87, agreeing with the original study.

The classifier found 98,603 images with good quality from a total of 175,709 fundus images, of which 49,903 were from the right eye and 48,700 from the left eye. These images were derived from ~45,000 participants with different ancestries and included individuals with both or one eye examined at least one time. In the case of those participants that had two good quality images from one eye, following analyses are completed using the images obtained at the first examination.

Besides classification, the classifier returns an imaging quality score (IQS) based on the distance of an image from the classification boundary computed at the training phase of the SVM. We retrieved IQS using the score parameter in the prediction function running in MATLAB 2018a. We thus quantify individually the reliability of each image being classified as bad and good image.

### Calculating fractal dimension

Retinal fractal dimension, D_f_, was computed from the binarized good-quality images using VAMPIRE software based on the multifractal analysis method^70^. This process was parallelised using 12 cores and 10GB per core.

### Statistics and reproducibility

To compare left and right D_f_ values we used participants who had both eyes scanned at the same UKBB examination and whose images were classified as good quality. 39,659 participants met these criteria. Both D_f_ distributions were compared using a paired T-Test and by estimating the Pearson correlation with the SciPy package in python 3. We also fitted a linear regression using respectively left and right D_f_ as dependent and independent variables.

We estimated the Pearson correlation and the effect of 779 UKBB traits on D_f_ by fitting univariate linear regressions with each variable and using D_f_ as the dependent variable. This included 121 quantitative variables (such as age, height, and BMI) and 658 binary variables (such as sex, diagnosed myopia, and diagnosed hypertension) which were extracted as reported elsewhere in^30^. The effect of IQS was also analysed following this approach. In addition, we evaluated the IQS difference effect on D_f_ variability by fitting univariate linear regression using participants who had a good-quality image of both eyes scanned at the same UKBB examination. These analyses were completed using SciPy in python 3. Allied graphs were created using matplotlib and seaborn graphical packages in python 3.

### Genome-wide association studies

We included 38,811 and 38,017 individuals in the right and left GWAS, respectively, with a self-reported and genotyped confirmed unrelated white-British ancestry^71^. Unrelated individuals were selected using a 0.0442 threshold from UKBB data and a previous work that established unrelated UKB participants with a white British ancestry^30^. Variants included were autosomal SNPs present in the genotyping arrays employed by UKBB and from the UKBB imputation panel with HWE > 10^−6^, MAF > 5 × 10^−3^, call rate >0.9 in unrelated white British individuals (kinship < 0.0442) and imputation score >0.9 in the imputed SNPs. The number of total SNPs analysed after quality control was 9,275,849.

Following genotype-level QC, a linear regression model was used to analyse the association of each SNP genotype with D_f_ using PLINK v2.0. We assumed an additive genetic model, adjusting for age at examination, sex, IQS, assessment centre, the first 10 genomic principal components and genotyping batch. In addition, we included hair and skin colour as covariates to control for the influence of skin and eye colour on the fundus image colour, which can affect image segmentation and D_f_ calculation. Hair colour replaced eye colour as the latter was not recorded during UKBB assessments, and it has a similar genetic control to eye pigmentation. Besides, we performed an additional GWAS including a polygenic risk score (PRS) for eye colour to assess its influence on our GWAS results. This PRS derives from an eye colour GWA study that defines it quantitatively (i.e., 1 = blue or grey, 2 = green, 3 = hazel, and 4 = brown) completed by Lona-Durazo et al. using the CanPath cohort, which includes ~5000 participants with European ancestry^29^. We estimated this PRS for each participant by extracting those independent genetic variants with a *P*-value < 5 × 10^−8^ from the summary statistics and applying linear regression to the effects of these SNPs and the genotypes of our UKBB participants (Supplementary Table 10). We then included this PRS as a covariate in an additional GWAS. Supplementary Figure 8 demonstrates that the results of these GWAS are analogous to those of GWAS including both skin and hair colour.

Furthermore, we completed a supplementary mean D_f_ GWAS using those participants with white British ancestry from both left and right eye populations. We decided that mean Df was calculated only on those participants whose image quality score for both eyes was within 3SD from the population mean. If this condition was not met, we used the Df measure from the eye with the highest IQS or the available measure. This left us with a sample size of 39,799 participants.

QQ plots were generated using the R package qqman and ggplot2, and Manhattan plots and GWAS comparisons plots were generated using Matplotlib and seaborn libraries in python 3.

We completed a PheWAS to assess whether D_f_ loci have a significant effect on other traits. To this end, we searched D_f_-associated SNPs in GWASCatalog^31^ and GeneATLAS^30^. GWAScatalog contains hundreds of GWAS performed in different traits and populations and it constantly updates new GWAS to its database. GeneATLAS contains GWAS summary statistics for 778 UKBB traits and diseases using individuals from European ancestry from UKBB. These genetic variants have a *P*-value smaller than 5 × 10^−8^ on the trait in order to assume a strong association common to D_f_.

### GWAS and meta-analysis of Df loci across UKBB ancestries

We performed additional GWAS including UKBB participants with European non-British (n_left_ = 4340 and n_right_ = 4288), Asian (n_left_ = 562 and n_right_ = 568) and African ancestries (n_left_ = 498 and n_right_ = 509) following the aforementioned model and procedure.

The multi-ancestry GWAS comparison was completed with those significant and independent SNPs from the D_f_ GWAS including white British participants. We extracted the summary statistics of these SNPs from the Asian, African, and white-European GWAS and compared their effects across UKBB ancestries. Forest plots were carried out with Meta package in R 4.0 software.

### Association between D_f_ genetic risk score and D_f_ measures in the CLSA

We complemented our replication study with an association analysis using D_f_ measures and the genotypes from CLSA participants with a white European ancestry. The Canadian Longitudinal Study on Aging (CLSA) is a large, national, stratified, random sample of ~50,000 Canadians aged 45 to 85 years at the time of recruitment (2010–2015), followed until 2033 (or until death), which aims at investigating the associations between various risk factors and incidence of chronic diseases^72^. A subset of 30,000 participants (i.e., comprehensive subset) had physical examinations and biological specimen collection, including fundus photographs (1 for each eye) obtained using the Topcon TRC-NW8 non-mydriatic retinal camera. A total of 50,957 retinal photographs, from 25,717 CLSA participants, were analysed using VAMPIRE (Vascular Assessment and Measurement Platform for Images of the Retina) software version 3.1, to compute the image quality (good/moderate/poor) and the fractal dimension (D_f_) of the retinal vascular pattern. Participants with poor quality images for both eyes were excluded for subsequent analyses.

Among the comprehensive subset, 26,622 CLSA participants (with 93% of Europeans) were successfully genotyped using the UK Biobank Array^71^. Quality control steps have been detailed elsewhere^73^. Briefly, phasing and imputation were conducted using the TOPMed reference panel^74^ at the University of Michigan Imputation Service^75^. We used the TOPMed reference panel version r^2^, and then pre-phased and imputed the genotype data using EAGLE2^76^ and Minimac^77^ respectively, for both autosomal and X chromosomes. Samples with low call rates (<95%), sex mismatches, or cryptic relatedness were removed. Imputed SNPs were excluded on the basis of HWE > 10^−6^, MAF > 1 × 10^−4^, call rate >0.9, and imputation quality (imputation score <0.6).

A total of 19 independent genetic variants significantly associated with D_f_ in the UKB were selected to calculate a genetic risk score (GRS) (Supplementary Table 11). CLSA Individual’s risk score consisted in the sum of each SNP dosage weighted by each SNP-D_f_ association coefficient given in D_f_ unit per effect allele. A linear regression was performed to estimate the association between FD measures and D_f_ GRS in 16,205 CLSA participants, with at least one retinal image of moderate or good quality of either side, and suitable genetic material. Models were adjusted for the 20 first principal components.

### Genetic correlation and heritability estimation

To investigate the shared genetic signal between D_f_ and associated traits, we estimated their genome-wide genetic correlation. For this purpose, we obtained the GWAS summary statistics of traits of interest to our study from GeneATLAS and the eye colour study^29^. These calculations were computed with LD Score^43^, a toolbox that estimates genetic correlation using GWAS summary statistics considering possible inflation caused by SNPs in linkage disequilibrium (LD). To ascertain the LD blocks within each variant, the software uses the 1000 Genomes panel as reference. Heatmaps were created with the genetic correlation estimate using the seaborn library in python 3.

LD Score was also used to calculate the SNP heritability of both eyes’ D_f_. In this case, the software uses the reference map and the GWAS summary statistics to estimate the fraction of D_f_ variance explained by the SNPs’ additive effect.

### Mendelian randomization

To infer the causality between the shared genetic basis of CAD, MI, hypertension and D_f_, we performed a Mendelian randomization analysis. For this procedure, we extracted the summary statistics of MI, hypertension, and CAD from GeneATLAS. We next selected for each cardiovascular condition separately those SNPs with a *P*-value < 5 × 10^−08^, and MAF > 0.01. We then selected those independent SNPs which were not palindromic by clumping these regions in windows of 10,000 kb and applying a r^2^ < 0.001 and a significance of 0.99 thresholds. The effect and the significance of these variants were also extracted from D_f_ GWAS summary statistics. We then estimated the causal effect of these genetic variants through different methods (inverse-variance weighted regression, Egger’s regression, and Maximum likelihood) to analyse whether using different scenarios could better characterise the causality. This process was completed with TwoSamplesMR package in R 4.0^78^. This package applies a quality control and a sensitivity analysis to evaluate the presence of palindromic SNPs, pleiotropy and heterogeneity which might influence the results of the study.

### Fine-mapping

Fine-mapping of significant D_f_ SNPs was completed with SusieR v.0.11.42 R package^79^. For each significant variant locus, we selected those variants that were located within 1 Mbp window at each side and estimated the correlation matrix among them with plink v1.9. Next, we ran the Susie_rss function with the Z-score from D_f_ GWAS and the correlation matrix of the previously selected variants. We ascertained that each credible set must have a coverage >0.95 and a minimum and median correlation coefficient (purity) of *r* = 0.1 and 0.5, respectively.

### Transcription factor binding sites prediction

The identification of variants with strong evidence to disrupt TF binding activity based on position probability matrices (PPM) was carried out with the R library motifbreakR v2.2.0^80^. For the TFBS we used default settings except the *P*-value threshold to declare TF binding site matching either of the allelic configurations, which was set to 5 × 10^−04^, and the relative entropy scoring method set to information content algorithm (method = ic) as performed in^81^. MotifDb and motifbreakR_motif were the selected databases of TF motifs which contain 14 public collections (including JASPAR, HOCOMOCO, ENCODE, HOMER and FactorBook) to perform this analysis. We calculated accurate *P*-values for both reference and alternative alleles by implementing calculatePvalue() function. We investigated those TFBS motifs with a *P-*value < 0.001 in both alleles and an absolute allelic score difference > 1.5.

Protein–protein interaction networks analyses were completed with those TF that bind at significant TFBS and the regulated genes located within a 150 kb window using DAVID^82^ and STRING^83^ software. We considered associated pathways those with an FDR and Bonferroni correction < 0.001.

### Development of MI predictive model

We used a subset of the UKBB data for MI model training and evaluation. We extracted white British UKBB participants who had good-quality images and a MI event after UKBB recruitment. MI events were defined in UKBB as a participant self-reporting MI at first repeated assessment visit [code 1075 from UKBB data field 20002] and MI hospitalizations identified using ICD10 codes [codes I21.1, I21.2, I21.3, I21.4, I21.9, I22, I22.0, I22.1, I22.8, I22.9, I23, I23.0, I23.1, I23.2, I23.3, I23.4, I23.5, I23.6, I23.8, I24.1, and I25.2 from UKBB data field 41204 and 41202]. The UKBB team previously validated this MI extraction algorithm and reported a minimum precision of 75%^84^. To define incident cases occurring after UKBB recruitment we used the date of the MI event [UKBB algorithmically defined MI event date from data field 42000] and the approximate period when participants underwent the ophthalmic examination, resulting in 526 incident cases. We randomly selected an equal number of age-matched participants with good-quality images of both eyes no cardiovascular event within the CAD spectrum and no known risk factor (e.g., hypertension, and family history of heart disease). This match was completed using the age range of the cases (i.e. 45–61 years) and constricting the random selection of controls to these ages.

Our MI predictive model uses age at baseline, sex, systolic blood pressure, smoking status, BMI, and a polygenic risk score for CAD and D_f_ of both eyes separately as features in a classification algorithm. We chose a random forest classifier algorithm allowing both non-linear associations between outcome and variables as well as inter-variable interaction in the model. Permutation-based feature importance scores^85^ were extracted in the modelling phase to assess the effect of each variable in the random forest construction using the *feature_importances_* function from the scikit-learn package. Given the influence of IQS on D_f_, we trained an additional model replacing D_f_ to D_f_ adjusted by IQS to assess the existence of major differences in the model’s performance. We also tested whether introducing just one eye D_f_ in the model implied major differences in its performance.

We then extracted the information of the established risk variables, that is, age, sex, SBP, BMI, and smoking status, for the population of study using the curated phenotypes from UK Biobank July 2017 release^30^ We extracted controls only considering those UKBB participants with no missing data and both D_f_ measures, as the majority of individuals with a D_f_ measure were healthy. 56 MI cases had a missing D_f_ measure from one eye. In these cases, we did not predict the missing value and only used the available Df measure.

To evaluate the performance of the predictive model, we reproduced SCORE with this MI dataset. SCORE uses age, sex, systolic blood pressure, smoking status, and BMI as input variables for logistic regression, with quantitative variables being discretized using healthcare guidelines^8^. We then assessed each model’s performance by using internal 10-fold cross-validation and computing its AUC, precision, and recall. We used the same data partitions across SCORE and our MI models. A Wilcoxon signed-rank test was completed across all the trained models to evaluate the significance of the AUC differences.

We used Kaplan–Meier curves to assess the difference in survival rate difference between patients with high and low predicted MI probability, dichotomised at a probability of 0.5. This probability was obtained with our top-performing MI model. A Log-rank test was completed to evaluate the difference between these groups’ curves.

We investigated the sources of improvement of our MI model compared to the SCORE model through an ablation study. The model differs from SCORE in four key aspects: (1) introducing D_f_, (2) the use of not-discretized quantitative variables, (3) using Random Forest instead of logistic regression, and (4) introducing PRS_CAD_. This ablation study consisted of assessing the performance of a modified version of SCORE through its AUC, recall and precision. These modifications included all the possible independent combinations across these alterations.

This part of the study was written in Python 3.5.7 using the sci-kit-learn, NumPy and Pandas packages. ROC curves were plotted using the predicted MI probability from each model using the ROCurve plot package in R 4.0. Both Kaplan–Meier curves and the Log-rank test were completed with the lifelines Python package.

### Estimating CAD polygenic risk score

PRS_CAD_ derives from the CARDIoGRAMplusC4D Consortium^21^ which is one of the largest completed CAD meta-analyses. This study does not include UKBB data, but it is developed with multiple CAD databases with different ancestries to better characterise the genetic control of this outcome. We estimated PRS_CAD_ for each participant in the MI dataset by using PRSice-2 software^86^, the summary statistics of the meta-analysis, and the genotypes of this MI dataset. We then included this PRS as a variable in our MI predictive model.

### Reporting summary

Further information on research design is available in the Nature Portfolio Reporting Summary linked to this article.

## Supplementary information

Bernabeu_Peer Review File

Supplementary Information-New

Description of Additional Supplementary Data

Supplementary Data 1

Supplementary Data 2

Reporting Summary

## Data Availability

The authors declare that the data supporting the findings of the present study are available within the paper and its supplementary information files. The fractal dimension GWAS summary statistics of all fitted models are openly available from the University of Edinburgh DataShare repository within the following collection: https://datashare.ed.ac.uk/handle/10283/4794. Data are available from the Canadian Longitudinal Study on Aging (www.clsa-elcv.ca) for researchers who meet the criteria for access to de-identified CLSA data.

## Code availability

The authors declare that the customised code for the myocardial infarction predictive model is available within the following GitHub repository: //github.com/Anavillaplana/MI_risk_prediction.
