# Supplementary material for: Fine-mapping of retinal vascular complexity loci identifies Notch regulation as a shared mechanism with myocardial infarction outcomes
Source: Commun Biol. 2023 May 15;6:523. doi: 10.1038/s42003-023-04836-9 (PMC10185685; doi:10.1038/s42003-023-04836-9)
Supplement: Supplementary file 2 — Supplementary Information-New [file 42003_2023_4836_MOESM2_ESM.pdf]

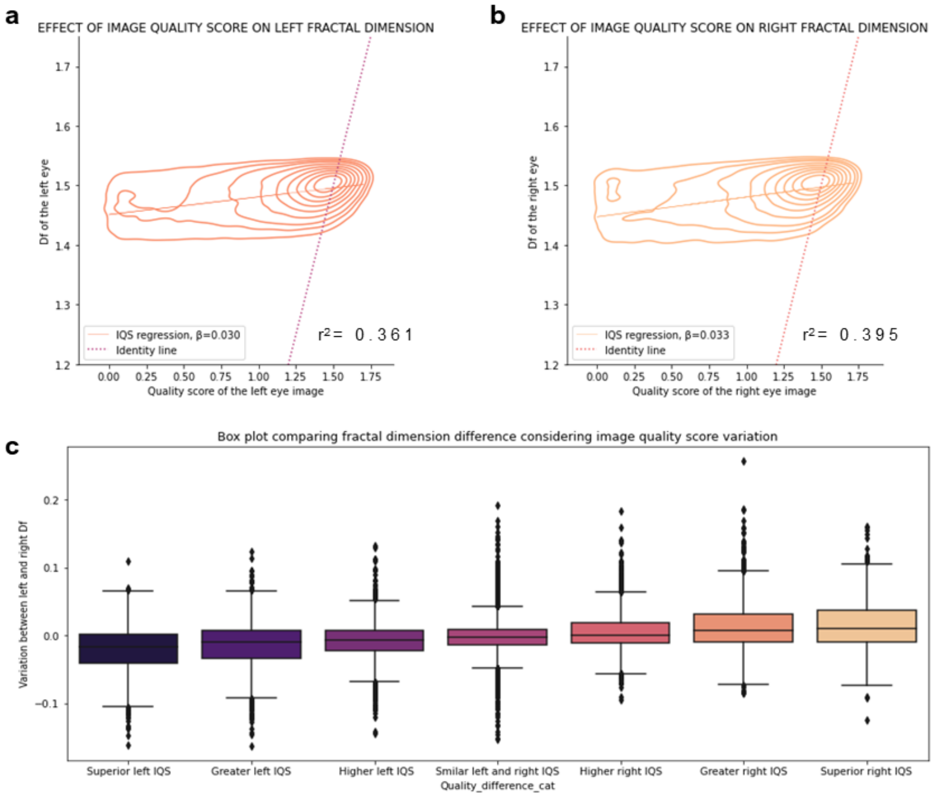

Supplementary Figure 1: Effect between image quality score (IQS) and fractal dimension. The contour plot evidences the joint distribution of **a** left and **b** right fractal dimension and image quality score. **C** The box plot illustrates the interocular fractal dimension difference at multiple IQS variation cases.

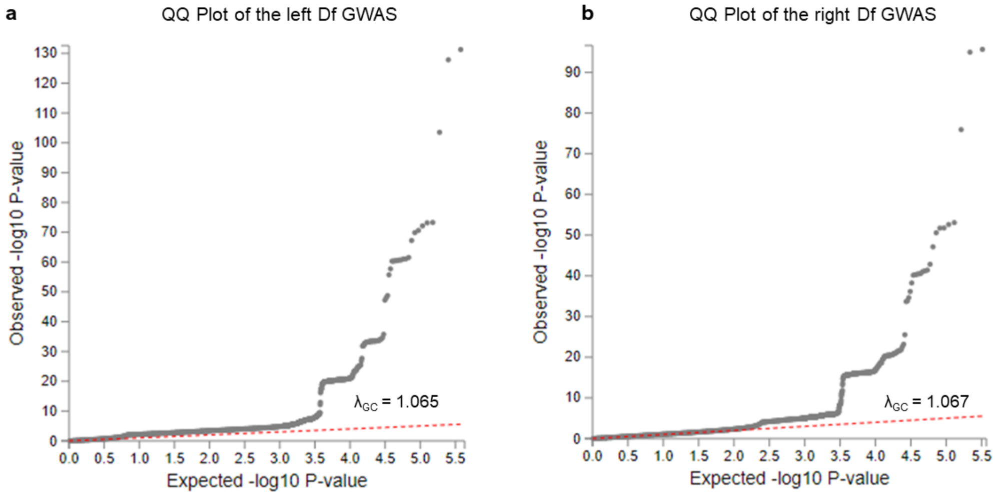

Supplementary Figure 2: QQ plots for the GWAS. These plots illustrate the expected vs observed -Log(P-value) comparison obtained in **a** the left and **b** right Df GWAS.

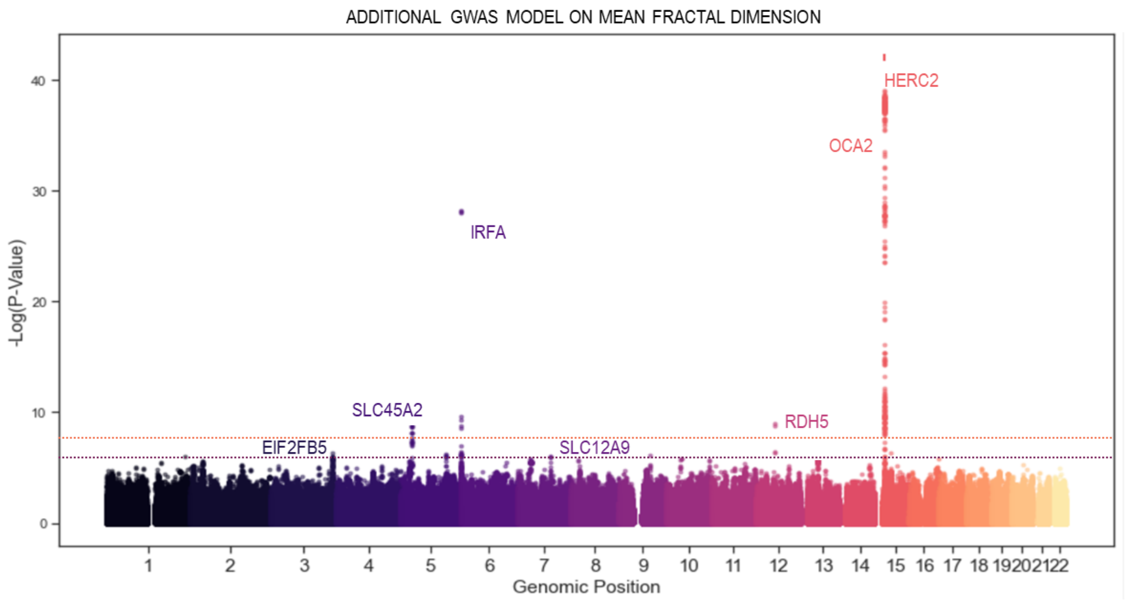

Supplementary Figure 3: Manhattan plot of mean Df GWAS. SNPs with a –Log10(P)<50 are included in this plot for clarity.

This additional D_f GWAS had a genomic inflation of 1.075, which indicates an adequate control of genomic inflation. The Manhattan plot of Supplementary Fig. 7 evidences that the SNPs associations are equivalent to those from eye-specific analysis.

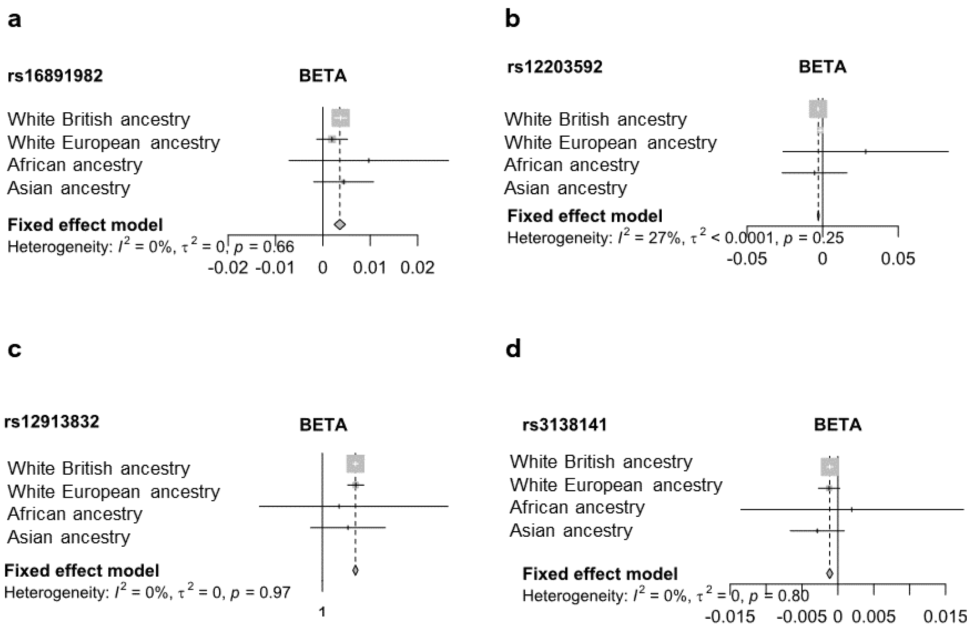

Supplementary Figure 4: Forest plots of Df-associated SNPs. These illustrate the effect of significant genetic variants (**a** rs16891982 **b** rs12203592 **c** rs12913832 **d** rs3138141) across UKB ancestries.

a

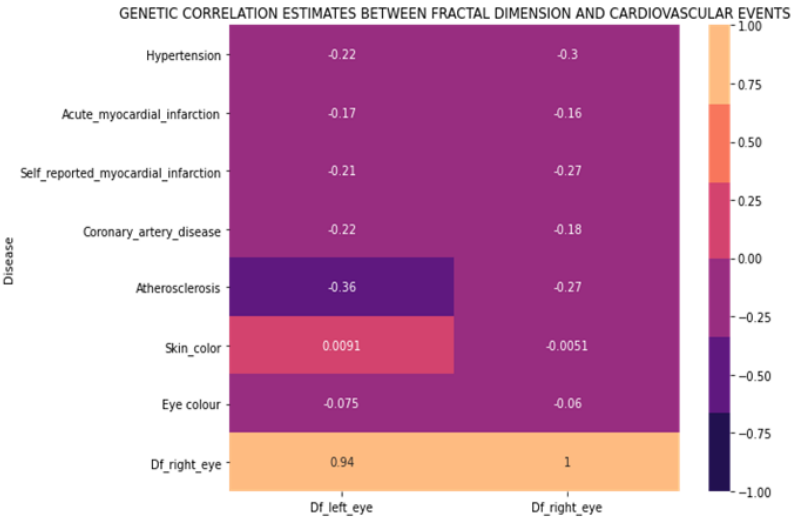

b

Correlated trait	LEFT EYE			RIGHT EYE		
	r_g^2	SE	P value	r_g^2	SE	P value
Hypertension	-0.2229	0.0534	3.0261e-05	-0.3020	0. 0659	4.52e-06
Acute myocardial infarction	-0.1717	0.0809	0.0801	-0.1585	0.1075	0.0308
Self reported acute myocardial infarction	-0.2071	0. 0754	0.006	-0.2663	0. 0982	0.0067
Coronary artery disease	-0.2214	0.0591	1.7854e-04	-0.1776	0. 0795	0.025
Atherosclerosis	-0.3585	0.1819	0.084	-0.2668	0.2100	0.051
Skin colour	0.0091	0.0536	0.8647	-0.0051	0. 0568	0.9282
Eye colour	-0.0751	0.0334	0.6372	-0.0598	0.0271	0.7658
Right Fractal dimension	0.9468	0.0962	1.75E-26	-		-

Supplementary Figure 5: Genetic correlations between fractal dimension and associated traits. a Heatmap illustrating the direction and percentage of shared genomic regions, also indicated by the number. **b** Table describing genetic correlation estimates and its P-value

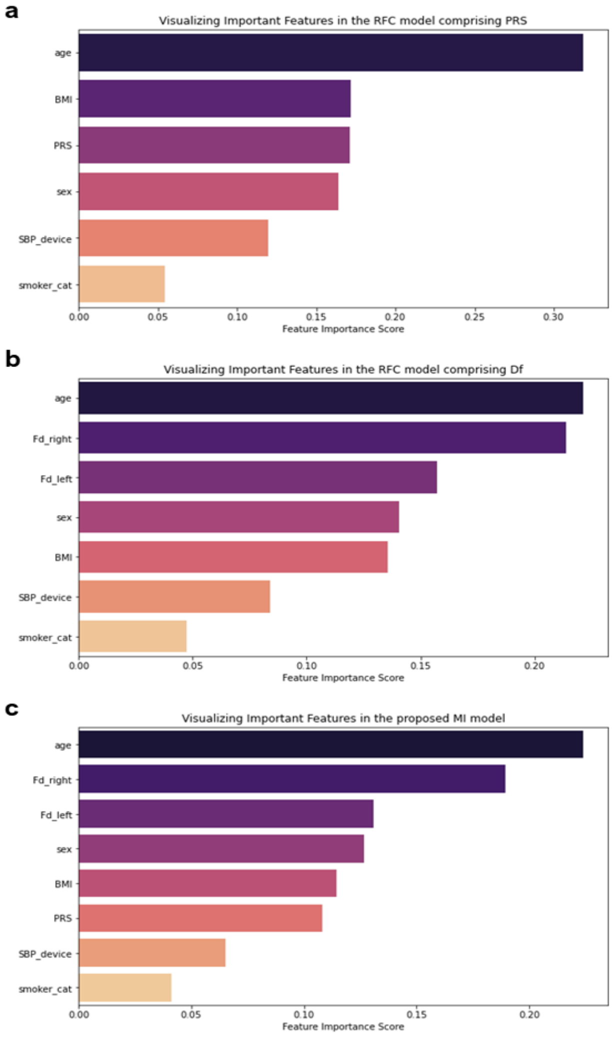

Supplementary Figure 6: Feature importance score of the MI models based on a random forest classifier. SBP device: Systolic blood pressure measured at baseline UKB assessment using their automatic device. PRS: CAD polygenic risk score based on CARdioGRAM consortium. BMI: basal muscular index. Fd_left and Fd_right: measures of left and right fractal dimension, respectively. Smoker cat: participants who are current smokers at baseline examination.

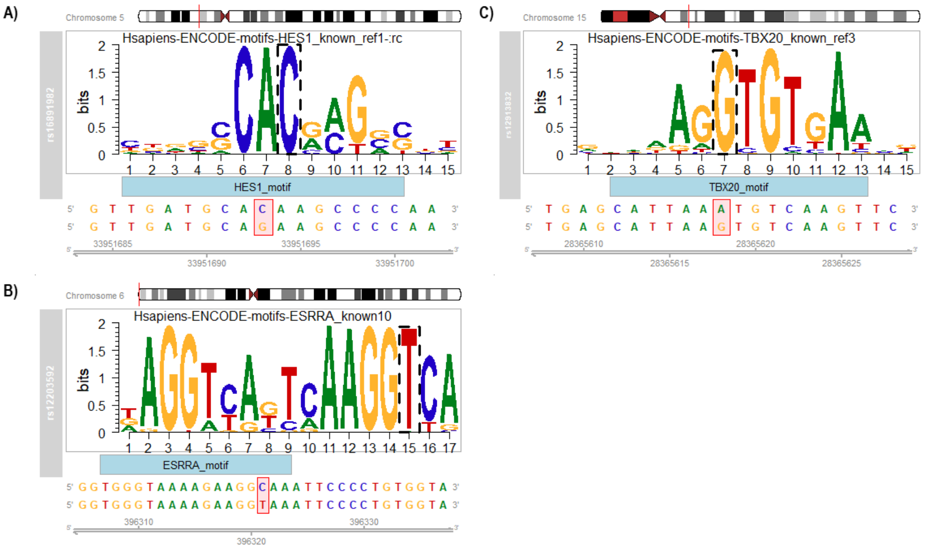

Supplementary Figure 7: Common activity alleles in HES1, TBX20, and ESRRA TFBS.

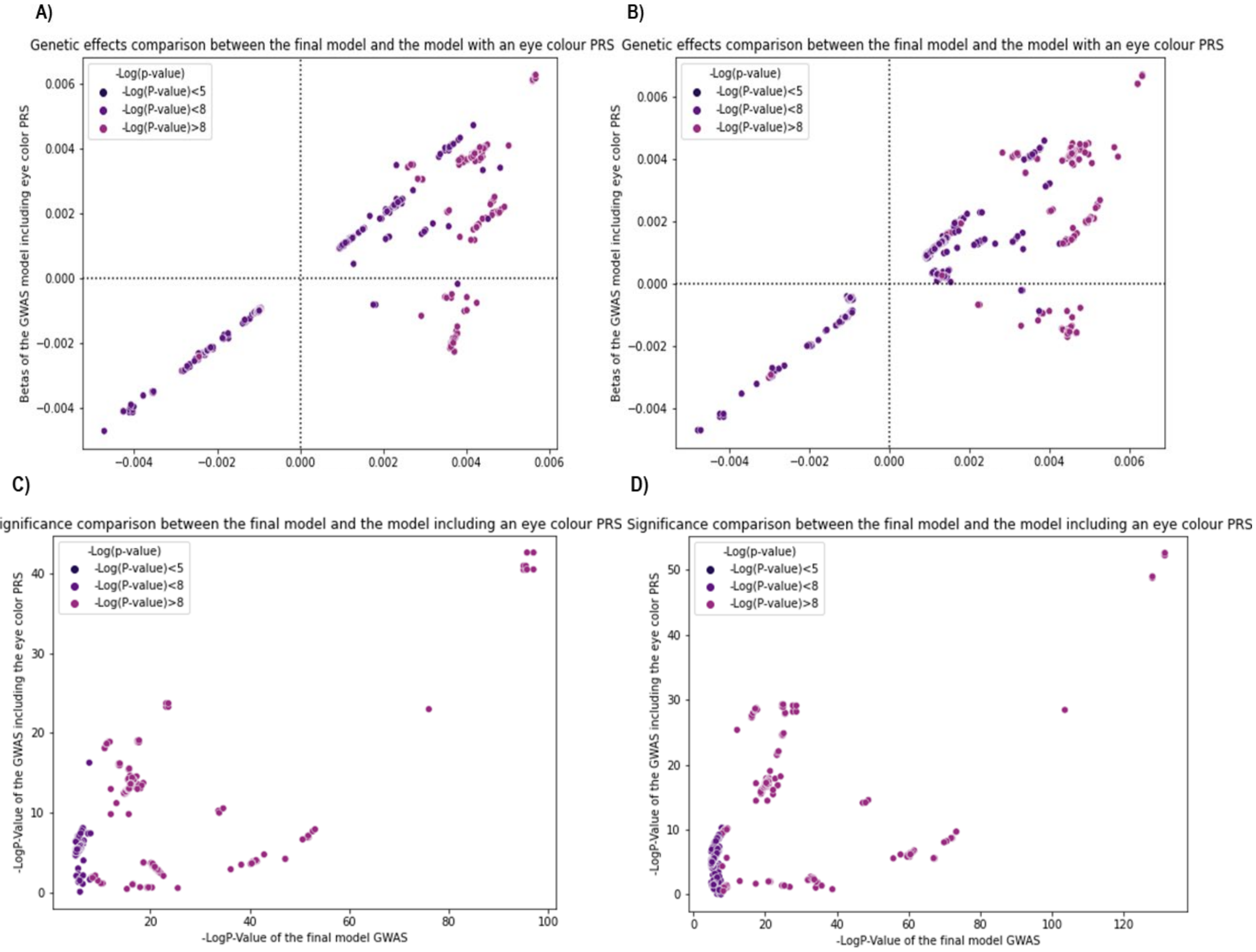

Supplementary Figure 8: Left and right eye GWAS results comparisons between the final model and this model including an eye colour PRS. SNPs with a –Log10(P)=4.5 are only included in these figures for clarity.

Supplementary Table 1: Fine-mapping results. Includes credible D_f set, its set size, posterior inclusion probability (PIP), Log (P-value) of PIP, the mean and median absolute correlation between the SNPs at each credible set.

CHR	SNP	Putative variant	Size of the credible set	SNPs of the credible set	Mean absolute correlation	Median absolute correlation	Log(P-Value)	PIP
3	rs73175105	Yes	1 SNP	-	1	1	2.337	0.955
5	rs16891982	Yes	2 SNP	rs28777	0.816	0.816	4.38	0.991
6	rs12203592	Yes	1 SNP	-	1	1	18.315	1.0
6	rs3778607	Yes	1 SNP	-	1	1	3.152	1.0
6	rs9375805	Yes	1 SNP	-	1	1	2.211	0.996
7	rs80308281	No	1 SNP	-	1	1	0.4	0.991
12	rs6018400	Yes	1SNP	-	1	1	1.95	0.95
15	rs12913832	Yes	2 SNP	rs1129038	0.986	0.986	74.254	0.975
15	rs72714116	Yes	1 SNP	-	1	1	2.296	0.979
15	rs75159657	Yes	1 SNP	-	1	1	1.779	1.0
15	rs58843292	Yes	4 SNP	rs8028689<br>rs6158505<br>rs2240204	0.803	0.803	2.503	0.986
21	rs73226964	Yes	1 SNP	-	1	1	7.78	0.991

Supplementary Table 2: GWAS summary statistics. Includes the MAF, SNP effect, SD and -Log(P-value) of all significant SNPs for both eyes. The nearest gene, its association with ocular or non-ocular traits it is also included.

SNP	CHR	MAF	LEFT EYE			RIGHT EYE			Nearest gene	Ocular association	Non-ocular association
			BETA	SD	-Log (P-value)	BETA	SD	-Log (P-value)			
rs73175105	3	0.23	-1.01E-04	3.60E-04	5.22	-1.83E-03	3.33E-04	5.33	*EIF2B5*		Inflammation and platelet count
rs16891982	5	0.024	3.75E-03	6.59E-04	7.93	3.53E-03	6.94E-04	6.46	*SLC45A2*		Skin, hair and eye colour and pigmentation disease
rs12203592	6	0.22	-2.31E-03	2.68E-04	28.67	-2.85E-03	2.80E-04	23.62	*IRF4*	Refractive error	Skin, hair colour, eye colour and disease and lymphocyte and leukocyte count.
rs56108400	12	0.24	-1.07E-03	2.37E-04	5.17	-1.06E-03	2.48E-04	5.72	*RDH5/ ORMDL2*	Macular thickness, AMD, retinitis pigmentosa, disorders of the lens, cataract, ocular muscle, myopia and retinal detachment	
rs12913832	15	0.22	6.34E-03	2.58E-04	131.28	5.65E-03	2.71E-04	96.97	*HERC2*	Cataract, retinal arterial and venular width and tortuosity, visual acuity, AMD and IOP	Pulse pressure and hair, skin and eye colour
rs72714116	15	0.15	3.33E-03	5.99E-04	27.07	4.20E-03	6.35E-04	51.76	*OCA2*	Cataract, IOP, lens disorders and Glaucoma	Hair, skin, and eye colour
rs73226964	21	0.02	-4.21E-03	7.48E-04	4.63	-4.00E-03	7.75E-04	6.63	*AGPAT3*		RBC count, eosinophil percentage, GFR diabetes miellitus, and disorders of the arteries.

Supplementary Table 3: Comparison between Zekavat et al. GWAS and this study. Includes reported genetic variants

SNP	MAF	NEAREST GENE	Zekavat et al[5]		This study	
			BETA	-Log (P-value)	BETA	-Log (P-value)
rs16891982	0.024	*SLC45A2*	0.17	13	3.53E-03	6.46
rs12203592	0.22	*IRF4*	-0.05	13	-2.85E-03	23.62
rs80308281	0.005	*SLC12A9*	-0.28	11	-7.19E-03	6.88
rs12913832	0.22	*HERC2*	0.15	78	5.65E-03	96.97
rs72714116	0.15	*OCA2*	0.12	43	4.61E-03	51.76

Supplementary Table 4: Mendelian randomization results. Includes the heterogeneity and pleiotropy test as well as the statistic and P-value of the MR methods used for both cardiovascular outcomes in both eyes.

	Left Df						Right Df					
Cardiovascular event	Heterogeneity	Horizontal Pleiotropy	Inverse variance weighted	P-value	Maximum likelihood	P-value	Heterogeneity	Horizontal Pleiotropy	Inverse variance weighted	P-value	Maximum likelihood	P-value
Myocardial infarction	0.367	0.006	0.080 ±0.101	0.429	0.080 ±0.102	0.427	0.394	0.007	0.095 ±0.104	0.358	0.096 ±0.104	0.357
Mild Ischaemic heart disease	0.552	0.033	-0.064 ±0.122	0.600	-0.064 ±0.125	0.610	0.619	0.029	-0.074 ±0.049	0.533	-0.074 ±0.052	0.551

Supplementary Table 5: Transcription-factor binding site disruptiveness analysis. This table describes those TFBS with a strong disruptive effect and a P-Value<0.005 associated with credible D_f SNPs. It includes the score effect of both reference and alternative alleles (scoreRef and scoreAlt, respectively and its normalised estimate, pctRef and pctAlt) and the allelic difference between scores.

CHR	SNP	REF	ALT	Gene	TF Database	pctRef	pctAlt	scoreRef	scoreAlt	Refpvalue	Altpvalue	altPos	alleleDiff	effect
15	rs12913832	A	G	HDAC2	ENCODE-motif	0.933	0.794	11.136	9.497	4.58E-05	1.07E-03	1	-1.639	strong
5	rs16891982	C	G	HES1	ENCODE-motif	0.958	0.745	7.619	5.980	5.48E-05	1.05E-04	1	-1.639	strong
15	rs12913832	A	G	NR4A1	HOMER	0.901	0.765	10.937	9.321	7.18E-05	1.54E-03	1	-1.616	strong
15	rs12913832	A	G	NR4A2	ENCODE-motif	0.910	0.769	10.608	9.008	1.65E-05	6.91E-04	1	-1.599	strong
6	rs12203592	C	T	HIC1	ENCODE-motif	0.792	0.714	13.683	12.364	4.45E-05	4.40E-04	1	-1.318	strong
15	rs12913832	A	G	NR6A1	ENCODE-motif	0.794	0.716	13.265	11.998	4.26E-05	4.20E-04	1	-1.267	strong
12	rs117863863	A	G	NRF1	HOCOMOCO	0.804	0.681	7.709	6.579	2.54E-04	3.91E-03	1	-1.130	strong
3	rs73175105	T	G	HNF1A	HOMER	0.880	0.764	8.194	7.174	1.59E-05	4.74E-04	1	-1.020	strong
3	rs73175105	T	G	HMGA2	HOCOMOCO	0.905	0.841	11.123	10.345	5.99E-06	6.99E-05	1	-0.778	strong
3	rs73175105	T	G	HNF1B	HOCOMOCO	0.891	0.803	7.843	7.091	9.17E-06	1.93E-04	1	-0.752	strong
12	rs117863863	A	G	TP53	HOCOMOCO	0.728	0.672	9.611	8.905	6.99E-04	2.96E-03	1	-0.706	strong
6	rs12203592	C	T	NR1H	ENCODE-motif	0.765	0.813	11.845	12.568	2.75E-04	7.12E-05	1	0.723	strong
6	rs12203592	C	T	BARHL2	ENCODE-motif	0.827	0.945	7.441	8.455	5.54E-04	2.17E-05	1	1.014	strong
21	rs73227001	C	G	PAX3	HOMER	0.771	0.890	7.837	9.010	8.36E-04	2.46E-05	1	1.173	strong
6	rs12203592	C	T	BARHL1	ENCODE-motif	0.794	0.925	8.379	9.712	8.84E-04	2.67E-05	1	1.334	strong
15	rs12913832	A	G	TBX4	ENCODE-motif	0.719	0.832	9.161	10.518	8.22E-04	2.41E-05	1	1.357	strong
15	rs12913832	A	G	TBX5	ENCODE-motif	0.678	0.780	10.632	12.154	8.60E-04	3.71E-05	1	1.522	strong
15	rs12913832	A	G	TBX20	ENCODE-motif	0.732	0.893	7.427	8.992	3.30E-03	9.84E-05	1	1.565	strong
6	rs12203592	C	T	ESRRA	ENCODE-motif	0.681	0.765	13.081	14.664	6.13E-04	4.94E-05	1	1.583	strong
3	rs73175105	T	G	SIX1	HOMER	0.782	0.958	7.373	8.989	3.17E-03	7.83E-05	1	1.616	strong

We complemented our functional analysis with *in-silico* TFBS disruptiveness prediction of credible variants. We observed four credible D_f SNPs with a strong disruptive effect in 9 transcripts, which participate at different Notch signalling pathway stages. One possible mechanism is the alteration of *ESRRA* binding affinity, which influences *VEGFA* transcription. In-vitro and animal model studies indicate that *VEGFA* upregulation after an MI event activates the VEGF signalling pathway, which has a crosslink with the Notch pathway and increases its activity. Zhao et al suggest that *VEGFA* levels increase after 2h post-MI, which activates the angiogenic response in the injured tissue. Another

regulating Notch mechanism derives from *HES1* binding site affinity. *HES1* influences *MAML1* and *NOTCH1* expression and directly affect Notch activity. This *HES1*-mediated activity following an MI event has been widely investigated. Numerous animal models and in-vitro studies confirmed that greater *HES1* activity after the infarction results in Notch upregulation, which improves myocardial viability, regeneration, and survival rate. The last mechanism influencing Notch activity is mediated through *TBX20* binding site affinity, which plays a role in *TL3* transcription. Mutations and the presence of alternative alleles in this TF and its TFBS have been widely described in congenital heart disease and cardiovascular dysfunction, such as impaired QRS duration. Under a MI event, multiple studies indicate that *TL3* upregulation activates PI3K/Akt signalling pathway, a downstream process of Notch signalling pathway. PIK3/Akt activation results a cell viability improvement and cardiac recovery. Similarly, separate knockout mice studies for *ESRRA*, *HES1* and *TBX20* genes report a Notch downregulation, which concludes with a worse MI outcome. We hypothesize that the TF binding disruption caused by these genetic variants influence Notch activity and, in a case of MI, might have a cardioprotective effect as shown in aforementioned published studies. Thus, these analyses suggests that there is an intricate shared genetic basis between vascular complexity and MI and further in-vitro and in-vivo experiments are needed to characterise gene expression and regulation of retinal tissue to better understand its common genetic control.

Besides, the alleles that predict a stronger TFBS disruptiveness have a positive effect size on Df. We thus hypothesize that Df credible loci-Notch1 activity association might also influence the development of the retinal vascular system. Evidence suggests that Notch signalling pathway plays a key role in the development of the vascular system. It might be the case that the modulation of Notch activity through Df loci at development stages might alter the complexity of retinal vascular architecture. Nevertheless, further in-vivo and in-vitro experiments in healthy, developmental, and pathological conditions are required to expand our comprehension of the genetic architecture of the retinal vascular complexity.

Supplementary Table 6: Demographics of the MI cases and controls. This table describes for cases and controls the centrality and dispersion of the epidemiological variables included in the MI predictive model. P-value* refers to the T-test completed between these two groups to estimate its difference.

Variable	MI cases	Control cases	P-Value*
Age (years)	57.31±6.47	54.21±7.84	1.076e-39
Sex (N.Females/N.Males)	122/403	298/227	-
BMI	28.54±4.63	26.42±4.39	5.52e-14
SBP (mmHg)	142.03±20.20	135.37±18.01	2.21e-8
Current smokers	69	28	-
Right D_f	1.485±0.03	1.5±0.075	3.33e-15
Left D_f	1.485±0.03	1.494±0.036	1.97e-08
PRS_{CAD}	3.58±0.32	3.41±0.24	1.94e-06

Supplementary Table 7: Wilcoxon signed-rank test across the examined MI models. This table includes the Wilcoxon signed-rank sum test and the P-Value of all the comparisons between the models we trained in this study.

Models comparison	Wilcoxon signed rank sum test	P-Value*
SCORE VS RFC with D_f and PRS_{CAD}	1.0	0.0039
SCORE VS RFC with D_f	1.0	0.0039
SCORE VS RFC with PRS_{CAD}	6.0	0.027
RFC with D_f and PRS_{CAD} VS RFC with PRS_{CAD}	7.0	0.037
RFC with D_f and PRS_{CAD} VS RFC with D_f	25.0	0.846
RFC with PRS_{CAD} VS RFC with D_f	7.0	0.037

Supplementary Table 8: Additional MI models performance. These tables include the precision, recall and AUC for the variations in th0se supplementary MI models.

	MI		
Model	Precision	Recall	AUC
Random Forest including left Df	0.756±0.001	0.773±0.002	0.761±0.001
Random Forest including left Df and PRS_{CAD}	0.759±0.001	0.793±0.001	0.774±0.001
Random Forest including right Df	0.759±0.001	0.778±0.002	0.765±0.001
Random Forest including right Df and PRS_{CAD}	0.756±0.001	0.787±0.001	0.772±0.001
Random Forest including quality-adjusted Df	0.766±0.001	0.784±0.002	0.771±0.001
Random Forest including quality-adjusted Df and PRS_{CAD}	0.768±0.001	0.796±0.001	0.774±0.001

Supplementary Table 9: Ablation study of the MI models. These tables include the precision, recall and AUC for the variations in the model. * AUC estimates significantly different (Wilcoxson signed-rank test P-value<0.005) from the one obtained with the SCORE model. ** AUC estimates significantly different (Wilcoxson signed-rank test P-value<0.005) from the one obtained with the SCORE model and the one from our final model.

In our ablation study, we found three central elements ascertaining the superior accuracy of our model on distinguishing personalised MI risk in UKB. Firstly, the usage of continuous variables. Those models following this premise yield a greater performance than SCORE and its derivates (i.e., SCORE + D_f). We further investigated whether this

	MI		
Model	Precision	Recall	AUC
SCORE model[16]	0.705 ±0.00096	0.729 ±0.0019	0.711 ±0.0008
SCORE model and Df	0.705 ±0.00083	0.731 ±0.0021	0.711 ±0.00086
SCORE model and PRS_{CAD}	0.721 ±0.001	0.739 ±0.0016	0.723 ±0.0011
SCORE model and Df + PRS_{CAD}	0.718 ±0.0012	0.737 ±0.0019	0.727 ±0.0009
SCORE model using continuous variables	0.732 ±0.0014	0.752 ±0.0024	0.737 ±0.0013
SCORE model with continuous variables and Df**	0.728 ±0.0014	0.754 ±0.0023	0.735 ±0.0013
SCORE model with continuous variables and PRS_{CAD}**	0.753 ±0.00096	0.768±0.0013	0.747 ±0.00083
SCORE model with continuous variables and Df + PRS_{CAD}*	0.752 ±0.001	0.775±0.0013	0.759 ±0.00086
SCORE model using a RFC	0.711 ±0.0013	0.745 ±0.0007	0.719 ±0.001
SCORE model using a RFC and Df*	0.758 ±0.0013	0.765 ±0.0016	0.748 ±0.0013
SCORE model using a RFC and PRS_{CAD}	0.727 ±0.0012	0.737 ±0.0022	0.728 ±0.0011
SCORE model using a RFC and Df + PRS_{CAD}**	0.745 ±0.001	0.781 ±0.0014	0.752 ±0.0009
SCORE model using continuous variables and RFC**	0.733 ±0.0013	0.750 ±0.0024	0.738 ±0.0013
SCORE model using continuous variables, Df and RFC*	0.756 ±0.0008	0.778±0.0013	0.763 ±0.0011
SCORE model using continuous variables, PRS_{CAD} and RFC**	0.735 ±0.0010	0.756 ±0.0021	0.741±0.0012
SCORE model using continuous variables, Df, PRS_{CAD} and RFC*	**0.763±0.0016**	**0.788 ±0.0014**	**0.770 ±0.0013**

situation was reproduced in RFC-based classifiers. Our results show that these models achieve a higher AUC when compared with the ones that introduce age, BMI and SBP as discrete variables. Secondly, we observe that the presence of D_f and PRS_{CAD} in the predictive model significantly improves its performance, regardless of the classifier's algorithm. Finally, RFC-based models yield higher AUC, precision, and recall when compared with SCORE and all the completed transformations, implying that a non-linear

algorithm benefit individual MI prediction. Amongst these RFC classifiers, the one including both elements achieve the greatest performance, followed by a similar model excluding PRS_{CAD}.

Supplementary Table 10: List of selected SNPs used to calculate the eye colour PRS in UKB. These tables include SNP id from UKBB panel, the effect allele, its effect size and the SNP id in CanPath.

RSID	A1	A2	Effect size	STD Effect size
rs12203592	T	C	-0.16605	0.028599
rs1326779	C	T	0.140664	0.023747
rs8030798	C	T	0.155299	0.02305
rs2703950	G	C	0.19441	0.029025
rs2594935	A	G	0.22306	0.023114
rs735067	C	G	-0.17608	0.025829
rs8024968	T	C	0.546215	0.033751
rs72714116	T	C	0.701983	0.064527
rs56839008	T	C	0.762546	0.034904
rs77542847	A	C	0.726158	0.038979
rs4778138	G	A	0.805406	0.029666
rs1129038	T	C	-1.23263	0.023601
rs12913832	G	A	-1.25795	0.02373
rs75159657	T	C	0.973654	0.067099
rs61585051	T	C	0.974576	0.04382
rs116886437	A	C	-0.55021	0.083194
rs12591531	A	G	0.835912	0.037782
rs8028689	C	T	0.950556	0.041318
rs2240204	A	G	0.952132	0.042101
rs117743506	A	G	0.858504	0.060354
rs916977	C	T	-1.03934	0.027659
rs71467328	T	C	-0.46843	0.054214
rs1667394	T	C	-1.03748	0.027172
rs1635166	T	C	-0.96397	0.028632

Supplementary Table 11: List of selected SNPs used to calculate D_f GRS. These tables include SNP id from UKBB panel, the effect allele, its effect size and the SNP id in CLSA.

UKB_SNP_rsid	Effect allele	Effect size	Other allele	CLSA_SNP_position
rs7569637	T	0.0009407	G	chr2:8335055:G:T
rs7572436	A	0.00681038	G	chr2:8335209:G:A
rs4667727	G	-0.0052997	T	chr2:164038415:G:T
rs73175104	T	-0.0072647	A	chr3:176236774:A:T
rs35395	cT	0.00490958	C	chr5:33948484:T:C
rs16891982	C	0.0167344	G	chr5:33951588:C:G
rs12203592	T	-0.0356956	C	chr6:396321:C:T
rs62389423	A	-0.0003435	G	chr6:421281:G:A
rs62389424	A	-0.0004635	C	chr6:422631:C:A
rs3138142	T	-0.0059897	C	chr12:55721801:C:T
rs3138141	A	-0.0043255	C	chr12:55721994:C:A
rs72712694	C	-0.0063482	T	chr15:28062589:T:C
rs4778138	G	0.00104232	A	chr15:28090674:A:G
rs1129038	C	0.0233837	T	chr15:28111713:C:T
rs12593929	G	0.00200661	A	chr15:28114112:G:A
rs12913832	A	0.0815825	G	chr15:28120472:A:G
rs4911429	A	-0.0031963	G	chr20:34545749:G:A
rs6141488	G	-0.0023726	C	chr20:34604942:C:G
rs73227001	G	-0.0067415	C	chr21:44056356:C:G
