# Supplementary material for: Fine-mapping of retinal vascular complexity loci identifies Notch regulation as a shared mechanism with myocardial infarction outcomes
Source: Commun Biol. 2023 May 15;6:523. doi: 10.1038/s42003-023-04836-9 (PMC10185685; doi:10.1038/s42003-023-04836-9)
Supplement: Supplementary file 3 — Description of Additional Supplementary Data [file 42003_2023_4836_MOESM3_ESM.pdf]

Description of Additional Supplementary Files

File name: Supplementary Data 1

Description: The source data behind the graphs of Figure 2. Sheet 1 includes the Df data from the left eye and sheet 2 contains Df data from the right eye.

File name: Supplementary Data 2

Description: Summary statistics of UKBB traits. The table includes the linear regression effect, its standard deviation, P-value, Pearson correlation and its P-value
