# Supplementary material for: Fine-mapping of retinal vascular complexity loci identifies Notch regulation as a shared mechanism with myocardial infarction outcomes
Source: Commun Biol. 2023 May 15;6:523. doi: 10.1038/s42003-023-04836-9 (PMC10185685; doi:10.1038/s42003-023-04836-9)
Supplement: Supplementary file 6 — Reporting Summary [file 42003_2023_4836_MOESM6_ESM.pdf]

nature portfolio

Corresponding author(s): Miguel O. Bernabeu

Last updated by author(s): Mar 10, 2023

Reporting Summary

Nature Portfolio wishes to improve the reproducibility of the work that we publish. This form provides structure for consistency and transparency in reporting. For further information on Nature Portfolio policies, see our Editorial Policies and the Editorial Policy Checklist.

Statistics

For all statistical analyses, confirm that the following items are present in the figure legend, table legend, main text, or Methods section.

n/a	Confirmed	
☐	☒	The exact sample size (*n*) for each experimental group/condition, given as a discrete number and unit of measurement
☐	☒	A statement on whether measurements were taken from distinct samples or whether the same sample was measured repeatedly
☐	☒	The statistical test(s) used AND whether they are one- or two-sided<br>*Only common tests should be described solely by name; describe more complex techniques in the Methods section.*
☐	☒	A description of all covariates tested
☐	☒	A description of any assumptions or corrections, such as tests of normality and adjustment for multiple comparisons
☐	☒	A full description of the statistical parameters including central tendency (e.g. means) or other basic estimates (e.g. regression coefficient) AND variation (e.g. standard deviation) or associated estimates of uncertainty (e.g. confidence intervals)
☐	☒	For null hypothesis testing, the test statistic (e.g. *F*, *t*, *r*) with confidence intervals, effect sizes, degrees of freedom and *P* value noted<br>*Give P values as exact values whenever suitable.*
☒	☐	For Bayesian analysis, information on the choice of priors and Markov chain Monte Carlo settings
☒	☐	For hierarchical and complex designs, identification of the appropriate level for tests and full reporting of outcomes
☐	☒	Estimates of effect sizes (e.g. Cohen's *d*, Pearson's *r*), indicating how they were calculated

Our web collection on statistics for biologists contains articles on many of the points above.

Software and code

Policy information about availability of computer code

Data collection	We used VAMPIRE V3.1, which is available upon demand at https://vampire.computing.dundee.ac.uk/, to calculate fractal dimension and Matlab (v 2018a, available upon licensing at https://uk.mathworks.com/) to obtain image quality score from the imaging database.
Data analysis	We used PLINK (v1.9 and v2.0, freely available online at https://www.cog-genomics.org/plink/2.0/), LD Score Regression (v.1.0.1 freely available online at https://github.com/bulik/ldsc), Meta (v4.0 freely available at https://rdrr.io/cran/meta/man/meta-package.html), TwoSamplesMR (V 4.0 freely available at https://mrcieu.github.io/TwoSampleMR/), Susie (v 0.11.42 freely available at https://github.com/stephenslab/susieR), motifbreakR (v 2.2.0 freely available at https://github.com/Simon-Coetzee/motifBreakR), STRING (v 11.5 freely available at https://string-db.org/), DAVID (v 2022q2 freely available at https://david.ncifcrf.gov/), ROCR (V 1.0-11 freely available at https:// github.com/ipa-tys/ROCR/) and PRSice2 software(v 2.3.5 freely available at https://www.prsice.info/)

For manuscripts utilizing custom algorithms or software that are central to the research but not yet described in published literature, software must be made available to editors and reviewers. We strongly encourage code deposition in a community repository (e.g. GitHub). See the Nature Portfolio guidelines for submitting code & software for further information.

Data

Policy information about availability of data

All manuscripts must include a data availability statement. This statement should provide the following information, where applicable:

- Accession codes, unique identifiers, or web links for publicly available datasets
- A description of any restrictions on data availability
- For clinical datasets or third party data, please ensure that the statement adheres to our policy

This study has been conducted using the UK Biobank Resource under project 788 and the Canadian Longitudinal Study on Aging, CLSA dataset 1906019_McMaster_GPare_Baseline (Baseline Comprehensive (COM) version 4.2), under Application Number 1906019 . The GWAS comparison employed the available summary statistics from https://www.ahajournals.org/doi/10.1161/CIRCULATIONAHA.121.057709.

Human research participants

Policy information about studies involving human research participants and Sex and Gender in Research.

Reporting on sex and gender	This study included sex as a covariate for the genetic and predictive efforts. Sex was determined on self-reporting for most of the analyses, except for the GWAS where the self-reported sex needed to match that inferred from the genotypes.
Population characteristics	UK Biobank (https://www.ukbiobank.ac.uk) is a large multi-site cohort study that consists of 502,655 individuals aged between 40 and 69 years at baseline, recruited from 22 centres across the UK during 2006-2010. This study mainly considered 47,315 white British participants with a good quality fundus image which passed the genetic quality control tests.<br><br>The Canadian Longitudinal Study on Aging (CLSA) is a large, national, stratified, random sample of ~50,000 Canadians aged 45 to 85 years at the time of recruitment (2010-2015), followed until 2033 (or until death), which aims at investigating the associations between various risk factors and incidence of chronic diseases. A subset of 30,000 participants (ie, comprehensive subset) had physical examinations and biological specimen collection, including fundus photographs (1 for each eye). Participants with poor quality images for both eyes were excluded for subsequent analyses.
Recruitment	Data collection was completed by the UK Biobank employing the following recruitment guidelines: " Recruitment were via centrally coordinated identification and invitation from population-based registers (such as those held by the NHS) of potentially eligible people living within a reasonable traveling distance of an assessment centre (located around the UK). This central recruitment strategy will allow invitations to be targeted to enhance generalisability and to make allowance for the impact on participation rates of various factors (e.g. age, sex, ethnicity, socioeconomic status). Each assessment centre will aim to recruit as many as possible of the nearby target population during a period of about six months to one year (depending on the local population density and transport links). and will then be relocated in order to achieve recruitment across most of the UK".<br><br>Data collection was completed by CLSA employing the following guidelines: "Participation in the CLSA cohort is voluntary and all individuals provided written informed consent. ".The selection and recruitment process is detailed elsewhere, but in brief, a random sample of eligible households was contacted, and if an eligible individual in the household was identified, they were asked to provide their information to the CLSA in order to be contacted for recruitment. Those who responded by providing their contact information were considered pre-recruits. These pre-recruits were then contacted, and those who underwent all required baseline interviews and assessments and provided written informed consent were enrolled into the cohort. The participation rate into the CLSA was about 45% with an overall response rate of 10%.
Ethics oversight	The study was approved by the National Research Ethics Committee, reference 11/NW/0382, and informed consent was obtained from all UK Biobank participants as part of the recruitment and assessment process. This research has been conducted using the UK Biobank Resource under project 788.<br>The Canadian Longitudinal Study on Aging protocol was reviewed and approved by 13 research ethics boards across Canada. CLSA participants provided written informed consent as part of the recruitment and assessment process. This research has been conducted using the CLSA dataset 1906019_McMaster_GPare_Baseline (Baseline Comprehensive (COM) version 4.2), under Application Number 1906019. The CLSA is led by Drs. Parminder Raina, Christina Wolfson and Susan Kirkland.

Note that full information on the approval of the study protocol must also be provided in the manuscript.

Field-specific reporting

Please select the one below that is the best fit for your research. If you are not sure, read the appropriate sections before making your selection.

☒ Life sciences ☐ Behavioural & social sciences ☐ Ecological, evolutionary & environmental sciences

For a reference copy of the document with all sections, see nature.com/documents/nr-reporting-summary-flat.pdf

Life sciences study design

All studies must disclose on these points even when the disclosure is negative.

Sample size	This project is an analyses of the UK Biobank July 2017 release data. A total of 47,315 white British participants were considered for the main analysis. Validation of the main analysis was completed using phenotypic and genotyping data from 25,717 participants from the CLSA cohort. The predictive model presented in the last part of the study consisted of a data subset of this population, which included 1052 participants. Sample size for every analysis was determined by data availability.
Data exclusions	This study considered a total of 50,712 UKB participants that had at least one good quality fundus image. Main analyses were completed in 47,315 UKB unrelated white British participants after excluding individuals with genotyping outliars and had a missingness rate > 5% for the genetic variants that passed controls, participants whose self-reported sex did not match the inferred from their genotypes, and with no good quality images. Validation analysis were conducted with CLSA data, which included 27,727 participants after excluding samples with low call rates (<95%), sex mismatches, cryptic relatedness and poor quality images. These procedures ensured minimal confounding factors caused by poor phenotyping or genotyping quality.
Replication	GWAS replication was performed by comparison of our results to those published at https://www.ahajournals.org/doi/10.1161/CIRCULATIONAHA.121.057709. This comparison, detailed in the manuscript, essentially evaluated whether fractal dimension associated SNPs had consistent effect sizes and replicated its significant association. In addition, we collaborated with researchers using CLSA data with the aim of replicating our GWAS observations, which is comprehensively detailed in the manuscript.
Randomization	Not relevant as this study did no perform experimental analyses.
Blinding	The authors of this manuscript did not participate in the recruitment of any of the aforementioned cohorts, which served as basis for this study, and as such, were entirely blinded to data collection and processing.

Reporting for specific materials, systems and methods

We require information from authors about some types of materials, experimental systems and methods used in many studies. Here, indicate whether each material, system or method listed is relevant to your study. If you are not sure if a list item applies to your research, read the appropriate section before selecting a response.

Materials & experimental systems

n/a	Involved in the study	
☒	☐	Antibodies
☒	☐	Eukaryotic cell lines
☒	☐	Palaeontology and archaeology
☒	☐	Animals and other organisms
☒	☐	Clinical data
☒	☐	Dual use research of concern

Methods

n/a	Involved in the study	
☒	☐	ChIP-seq
☒	☐	Flow cytometry
☒	☐	MRI-based neuroimaging
