# Supplementary material for: Fine-mapping of retinal vascular complexity loci identifies Notch regulation as a shared mechanism with myocardial infarction outcomes
Source: Commun Biol. 2023 May 15;6:523. doi: 10.1038/s42003-023-04836-9 (PMC10185685; doi:10.1038/s42003-023-04836-9)
Supplement: Supplementary file 1 — Bernabeu_Peer Review File [file 42003_2023_4836_MOESM1_ESM.pdf]

nature portfolio

Peer Review File

Fine-mapping of retinal vascular complexity loci identifies Notch regulation as a shared mechanism with myocardial infarction outcomes.

Open Access This file is licensed under a Creative Commons Attribution 4.0 International License, which permits use, sharing, adaptation, distribution and reproduction in any medium or format, as long as you give appropriate credit to the original author(s) and the source, provide a link to the Creative Commons license, and indicate if changes were made. In the cases where the authors are anonymous, such as is the case for the reports of anonymous peer reviewers, author attribution should be to 'Anonymous Referee' followed by a clear attribution to the source work. The images or other third party material in this file are included in the article's Creative Commons license, unless indicated otherwise in a credit line to the material. If material is not included in the article's Creative Commons license and your intended use is not permitted by statutory regulation or exceeds the permitted use, you will need to obtain permission directly from the copyright holder. To view a copy of this license, visit http://creativecommons.org/licenses/by/4.0/.

Reviewers' comments:

Reviewer #1 (Remarks to the Author):

The authors present a GWAS of retinal vasculature fractal dimension and examine its genetic correlation with CAD and related traits. Ultimately they develop a prediction model for incident MI and argue a substantial improvement over previous prediction models. Notably, another group have recently published a cross-ancestry GWAS for fractal dimension, also in UK Biobank (Circulation. 2022;145:134–150). I have the following comments:

Major comments:
1. The rationale for analysing left and right eyes separately is not convincing to this reviewer. In Fig 2b, the left and right eye distributions look almost superimposed. The authors point out a statistically significant different between right and left eyes (line 125) but do not quantify this difference in the t-test. Asymmetry to some degree is expected (this exists for many ocular dimensions). For the GWAS, they are essentially modelling between-participant differences. To therefore assume that right eye values need to be compared separately to left eye values based on the above seems weak to me. Given the known power increases yielded by averaging data from both eyes, and given Fig 2b, and given the fact that the asymmetry clearly goes both ways (Fig 2d), I would think that taking the mean of right and left values would be the best approach.
2. If the authors do insist that there is a substantial systematic asymmetry in Df, then this would need to be explored further. Might this be related to asymmetric ocular disease (e.g. try excluding those with self-reported eye disease)? Or could this be related to refractive error (retinal Df is associated with refractive error and right eyes are known to be more myopic; https://www.aaojournal.org/article/S0161-6420(14)01037-9/fulltext) or maybe even amblyopia (Amblyopia is more common in the left eye (doi.org/10.1016/j.ajo.2010.01.040) and affects retinal FD; https://www.ncbi.nlm.nih.gov/pmc/articles/PMC4631015/)? Might this be related to the order in which eyes were examined? In particular, refractive error should be considered (and potentially adjusted for in the GWAS), and should feature in Supplementary Table 1.
3. The abstract suggests there were 9 significant loci, but the majority of these were only suggestive and not genome-wide significant
4. Have the authors convincingly demonstrated that the OCA2 and HERC2 loci are independent? In fact, the OCA2 locus SNP they report (rs916977) is in HERC2.
5. Given the sample size, wouldn't we expect more significant loci than just 3 (or arguably 2)? What does this mean? Is this not a very genetic trait, or are there issues with measurement?
6. The most convincing locus at OCA2/HERC2 is strongly associated with pigmentation. Might this still represent an artefact - might the image analysis software measure Df systematically differently in eyes of different pigmentation (which is not completely accounted for by simply adjusting for hair/skin colour)?
7. It is well-established that novel GWAS loci require replication in an independent cohort to have credibility. Given the availability of suitable cohorts (most notably the Canadian Longitudinal Study on Aging), I would suggest the authors seek to independently replicate their GWAS findings, as well as their prediction model findings.
8. In addition to genetic correlation, might Mendelian randomisation approaches be helpful too? At places in the manuscript, the authors do start referring to one parameter causing the other, yet a genetic correlation does not suggest causality, and certainly not direction.
9. I believe the authors have compared SCORE (age, sex, SBP and BMI) with 3 models incorporating either Df and/or the PRS. But the latter models also include smoking and there is no assessment of a model with SCORE + smoking. Clearly, smoking significantly affects retinal Df and significantly improves cardiovascular risk prediction tools (doi.org/10.1001/jamacardio.2021.4990). The authors should present a model of SCORE + smoking alone to make a true comparison of the added value of Df or PRS. The authors argue that the incremental benefit in discrimination between SCORE (age, sex, SBP and BMI) and a “random forest including Df” (age, sex, SBP, BMI, smoking and Df) is down to Df but smoking is in there too.

10. Prediction model reporting should adhere to TRIPOD guidelines. Many of the important items for reporting are missing in the manuscript. For example, how were missing data handled (e.g. missing covariables such as BP). Calibration as well as discrimination performance need to be presented.
11. The Kaplan-Meier survival analysis is unclear. Did the authors censor participants who died from a non-MI event? It is unclear why the KM curves drop only at yearly intervals when the linked outcome data provide more precise timings.

Minor comments:
12. Is the term “fine-mapping” used in the title reasonable? Perhaps there are different definitions, but this seemed like a straightforward GWAS to me without any fine-mapping of causal variants at each locus.
13. Why was there no cross-ancestry GWAS to maximise power?
14. Why was the Wilcoxon test used to compare models? These are nested models comparing SCORE to the addition of Df (and smoking). Why not just use a chi-squared likelihood ratio test, which is considered the gold standard by prediction model experts (see Frank Harrell Jr’s work on this)?
15. Please provide a clear rationale for comparing with SCORE rather than other prediction models. SCORE2 is developed and mostly used in European countries, QRISK3 is used in the UK. Both are far better than SCORE.
16. Please provide more detail and explanation for the matching. While the matching was only on age, there remains a reasonable difference in age post-matching (54.2 versus 57.3). However, there is no sex matching despite retinal Df and MI incidence differing in these groups (the same with hypertension and diabetes).
17. Please consider swapping precision and recall for sensitivity and specificity. The matched cohorts are balanced in numbers so the benefit of PR is limited and this metric is more familiar to computer science/information retrieval/engineering audiences. Also, given these are cardiovascular risk prediction models, comparison to other literature will mainly be through sensitivity/specificity.
18. Please can it be clarified whether the Df value in the risk prediction model is in some way adjusted by image quality? Apologies if this is somewhere in the methods but I could not find it, perhaps it can just be mentioned in the Results section near line 265.
19. Rather than SE, confidence intervals would be more informative in Table 3 and when presenting performance metrics on discrimination. It is easier to see overlap between models.
20. Please temper down any generalisability claims of the prediction model for MI. This was developed on a particular population with a small dataset who have a narrow sociodemographic profile.
21. Some of the terminology is a little loose - e.g. lines 89-90: the loci are at or near the genes (i.e. better to say the loci "at" or "near" SLC12A9 and RDH5, as the loci do not cover a whole gene and may not even be functionally related to the gene).
22. Line 169: references missing
23. Line 496: how were British individuals identified? How were unrelated participants selected?
24. Line 579: please explain the P-value threshold choice of 0.001
25. Line 595: could you please elaborate on “the approximate period when participants underwent the ophthalmic examination”

Reviewer #2 (Remarks to the Author):

In this paper the authors carry out a GWAS study on fractal dimension to further understand the genetics of Df, and also to understand it's link to CAD and MI. Overall, the paper is very well written and was interesting to read; however I am not sure how much novelty it introduces to the field.

I have a few comments below:
1. To clarify, the GWAS on Df was performed on 38,811 and 38,017 participants that had right and left Df measurements, were there overlapping people in this or were these 2 completely separate groups?

2. How significant (from a clinical stand point) is an increase in the AUC from 0.741 (SCORE) to 0.77? The ability to stratify patients by MI risk was very compelling.
3. Linking Df to MI via Notch and VEGF signaling is interesting. I did find the discussion about this to be confusing, are you suggesting that Notch signaling is being disrupted, leading to inhibition of Notch and VEGF signaling, which would lead to worse outcomes, or that the disruption is beneficial? Some more clarification around the discussion on this would be helpful.

Minor things:
1. Line 171, should read Fig 3d rather then 2d
2. In the extended discussion you have "W hypothesize" instead of "We"

We thank the reviewers for the feedback on our submitted manuscript. We have addressed here all the comments point by point and indicated where we have included such information in the updated version of the manuscript.

REVIEWER #1 (REMARKS TO THE AUTHOR):

MAJOR COMMENTS:

1. The rationale for analysing left and right eyes separately is not convincing to this reviewer. In Fig 2b, the left and right eye distributions look almost superimposed. The authors point out a statistically significant different between right and left eyes (line 125) but do not quantify this difference in the t-test. Asymmetry to some degree is expected (this exists for many ocular dimensions). For the GWAS, they are essentially modelling between-participant differences. To therefore assume that right eye values need to be compared separately to left eye values based on the above seems weak to me. Given the known power increases yielded by averaging data from both eyes, and given Fig 2b, and given the fact that the asymmetry clearly goes both ways (Fig 2d), I would think that taking the mean of right and left values would be the best approach.

We would like to thank the reviewer for the comment. We completed a supplementary GWAS including mean Df using those participants with white British ancestry. We decided that mean Df was calculated only on those participants whose image quality score for both eyes was within 3SD from the population median. If this condition was not met, we used the Df measure from the eye with the highest image quality score (IQS). Mean Df GWAS included 39,799 participants and had a genomic inflation of 1.075. The strongest associations of this GWAS (Manhattan plot c) were located in chromosome 15, followed by three independent loci at chromosomes 6, 5, and 12. We found no additional significant or suggestive SNP in this complementary GWAS, so our observations were consistent with those from the eye-specific GWASs presented in the previous version (see Table below). Genome-wide genetic correlation estimates between mean Df GWAS and eye-specific GWAS were 0.93 ± 0.03(P-Value=3.89e-201) and 0.89 ± 0.07 (P-Value=2.62e-38) for the right and left Df GWAS, respectively, suggesting that the SNPs effects of mean Df GWAS are comparable to that of left and right Df measures.

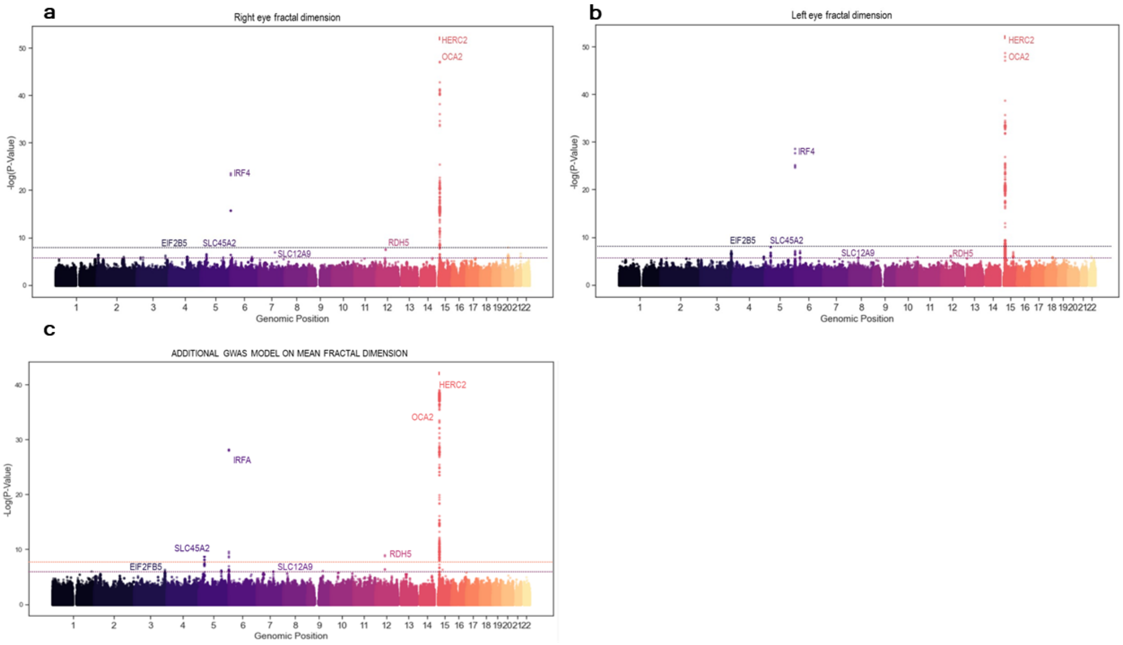

		RIGHT EYE		LEFT EYE		MEAN Df	
CHR	SNP	BETA (SD)	P-VALUE	BETA (SD)	P-VALUE	BETA (SD)	P-VALUE
3	rs73175105	-1.83E-03 (3.33E-04)	1E-06	-1.01E-03 (3.6E-04)	6E-06	-1.39E-03 (3.27E-04)	1.9E-06
5	rs16891982	3.53E-03 (6.94E-04)	3.44E-07	3.76E-03 (6.59E-04)	1.15E-08	4.15E-03 (6.01E-04)	4.77E-12
6	rs12203592	-2.85E-03 (2.82E-04)	6.59E-24	-2.99E-03 (2.7E-04)	2.28E-28	-3.19E-03 (2.44E-04)	6.28E-39
12	rs6018400	-1.06E-03 (2.48E-04)	1.09E-05	-1.07E-03 (2.37E-04)	7E-06	-1.08E-03 (2.14E-04)	4.79E-07
15	rs12913832	5.65E-03 (2.71E-04)	2.17E-97	6.34E-03 (2.58E-04)	5.24E-132	6.58E-03 (2.34E-04)	6.39E-173
15	rs72714116	4.20E-03 (6.35E-04)	3.71E-11	3.33E-03 (5.99E-04)	2.71E-08	3.23E-03 (5.50E-04)	3.97E-09
21	rs73226964	-4.01E-03 (7.75E-04)	2.32E-7	-1.44E-03 (7.49E-04)	5.34E-5	-2.69E-03 (6.70E-04)	5.8E-8

These observations from additional genetic analyses using mean Df have been included in the manuscript in line 163 as:

"Furthermore, an additional GWAS of mean D_f including participants from both left and right eye populations (see section "Methods") reported equivalent SNP associations to those from eye-specific populations (supplementary Fig. 7). The analogy between GWASs and genetic correlation estimates close to 1 between mean D_f and eye-specific GWAS (Mean D_f and right D_f: 0.93 ± 0.03, P-Value=3.89e-201; Mean D_f and left D_f: 0.89 ± 0.07, P-Value=2.62e-38) revealed that mean D_f GWAS was equivalent to those of left and right D_f measures."

In addition to our genetic study, we developed and evaluated a predictive model that included mean Df instead of eye-specific measures as well as the polygenic risk score (PRS). Mean Df, as in the previous analysis, was calculated if both eyes measures were available and had an IQS within 3SD from the median. Otherwise, we used the available Df measure with higher IQS. This model yielded reduced area under the curve (AUC), specificity, sensitivity and precision parameters than those versions including eye-specific measurements (see Table below). Nonetheless, this model version outperformed SCORE, which supports the claim that Df and the PRS have an additional predictive value not fully included in established risk factors in the study population.

	MI			
Model	Precision (95%CI)	Sensitivity (95%CI)	Specificity (95%CI)	AUC (95%CI)
SCORE model[16]	0.716 (0.664-0.741)	0.725 (0.691-0.767)	0.691 (0.652-0.731)	0.719 (0.681-0.737)
Random Forest including PRS_{CAD}	0.735 (0.708-0.782)	0.756 (0.726-0.801)	0.739 (0.702-0.777)	0.741 (0.725-0.775)*
Random Forest including both eye-specific Df	0.756 (0.732- 0.802)	0.778 (0.762-0.831)	0.758 (0.721-0.795)	0.763 (0.750-0.802)*
Random Forest including mean Df and PRS_{CAD}	0.733 (0.716-0.770)	0.779 (0.743-0.814)	0.756 (0.717-0.797)	0.748 (0.722- 0.773)*
Random Forest including Df and PRS_{CAD}	0.770 (0.734-0.805)	0.790 (0.757-0.826)	0.764 (0.728-0.800)	0.770 (0.751-0.802)*

These findings are described in line 276-278 of the updated manuscript as:

"Additional assessments in the definition of the Df variable indicated that the replacement of D_f measures with D_f adjusted by IQS, the introduction of one-eye D_f measurements in our MI model, or the use of mean D_f in our model yielded a comparable performance to the aforementioned ones (Supplementary Table 9)."

2. If the authors do insist that there is a substantial systematic asymmetry in Df, then this would need to be explored further. Might this be related to asymmetric ocular disease (e.g. try excluding those with self-reported eye disease)? Or could this be related to refractive error (retinal Df is associated with refractive error and right eyes are known to be more myopic; https://www.aaojournal.org/article/S0161-6420(14)01037-9/fulltext) or maybe even amblyopia (Amblyopia is more common in the left eye (doi.org/10.1016/j.ajo.2010.01.040) and affects retinal FD; https://www.ncbi.nlm.nih.gov/pmc/articles/PMC4631015/)? Might this be related to the order in which eyes were examined? In particular, refractive error should be considered (and potentially adjusted for in the GWAS), and should feature in Supplementary Table 1.

Our work accounted for interocular asymmetry because the hypothesis that there is a degree of symmetry between eyes remains *sub judice* which especially concerns retinal vascular measurements like Df. Previous studies clearly evidenced that there is a moderate Df between

eyes and discussed this matter in length (https://www.sciencedirect.com/science/article/pii/S135094621630091X and https://www.mdpi.com/2075-4418/12/6/1317). We complemented the systemic asymmetry evaluation in Df in our work by standardising the Df difference between left and right eye and comparing it with a normal distribution. This Df difference population had a mean of -0.0025 and the T statistic comparing this distribution with a normal distribution was -5.10 and the P-

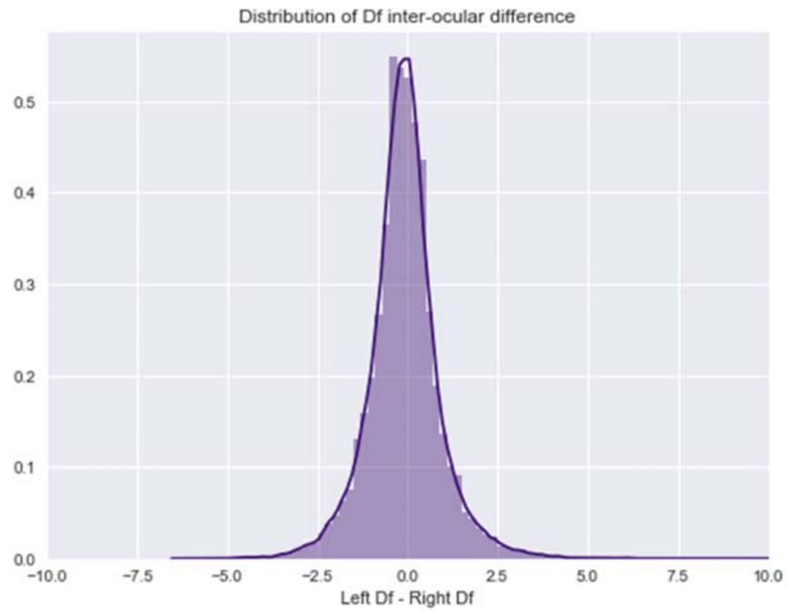

Value =2.79E-75 (see figure below), indicating that on average there is a difference between left and right eyes.

We also investigated the independent effect of refractive error and amblyopia of each eye in its corresponding Df measure and observed that only left eye refractive error had a significant effect on left Df. The effect size of this association was 0.00032 (P-Value=3.11x10-91), which is below the effect of previously associated traits, such as IQS, age, sex, ocular and cardiovascular conditions. Furthermore, as pointed out by the reviewer, the reported asymmetry could be influenced by the order in which eyes were examined as well as other unidentified factors. The UKBB protocol was to first analyse the right and then the left eye within 2 minutes of the ophthalmic assessment. We then speculate that the time constraint might have influenced the asymmetrical effect and considered that the completion of eye-specific analyses would reduce the potential effect of the examination bias. In this way, noise introduced by the ophthalmic examination that influenced the computation and analysis of Df is compensated for.

3. The abstract suggests there were 9 significant loci, but the majority of these were only suggestive and not genome-wide significant

We have modified the abstract to clearly indicate that our genetic analysis found 5 significantly associated loci across the completed GWAS and 4 loci below genome-wide significance that could have a suggestive effect of Df.

New abstract:

There is increasing evidence that the complexity of the retinal vasculature measured as fractal dimension, Df, might offer earlier insights into the progression of coronary artery disease

(CAD) before traditional biomarkers can be detected. This association could be partly explained by a common genetic basis; however, the genetic component of Df is poorly understood. **We present a genome-wide association study (GWAS) of 38,000 individuals with white British ancestry from the UK Biobank aimed to comprehensively study the genetic component of Df and analyse its relationship with CAD. We replicated 5 Df loci and found 4 additional loci with suggestive significance (P < 1e-05) to contribute to Df variation,** which previously were reported in retinal tortuosity and complexity, hypertension, and CAD studies. Significant negative genetic correlation estimates support the inverse relationship between Df and CAD, and between Df and myocardial infarction (MI), one of CAD's fatal outcomes. Fine-mapping of Df loci revealed Notch signalling regulatory variants supporting a shared mechanism with MI outcomes. We developed a predictive model for MI incident cases, recorded over a 10-year period following clinical and ophthalmic evaluation, combining clinical information, Df, and a CAD polygenic risk score. Internal cross-validation demonstrated a considerable improvement in the area under the curve (AUC) of our predictive model (AUC = 0.770±0.001) when comparing with an established risk model, SCORE, (AUC=0.741±0.002) and extensions thereof leveraging the PRS (AUC = 0.728±0.001). This evidences that Df provides risk information beyond demographic, lifestyle, and genetic risk factors. Our findings shed new light on the genetic basis of Df, unveiling a common control with MI, and highlighting the benefits of its application in individualised MI risk prediction.

4. Have the authors convincingly demonstrated that the OCA2 and HERC2 loci are independent? In fact, the OCA2 locus SNP they report (rs916977) is in HERC2.

We investigated the independence of reported loci by completing a fine-mapping analysis, which revealed that there are 4 credible SNP sets at chromosome 15 associated with Df (P-val < 0.05, see Table below). Two of these sets are located close to HERC2 and the remaining two are close to OCA2, with a PIP>0.95. This supports our claim that the signal at these loci are independent of each other.

We also changed the name of the SNP at OCA2 gene to *rs72714116* in the manuscript, as the SNP rs916977 is located at HERC2.

CHR	SNP	Nearest gene	Putative variant	Size of the credible set	SNPs of the credible set	PIP	P -Value
15	rs12913832	HERC2	Yes	2 SNP	rs1129038	0.975	5.57E-75
15	rs72714116	OCA2	Yes	1 SNP	-	0.979	0.005
15	rs75159657	HERC2	Yes	1 SNP	-	1.0	0.0166
15	rs58843292	OCA2	Yes	4 SNP	rs8028689<br>rs6158505<br>rs2240204	0.986	0.003

5. Given the sample size, wouldn't we expect more significant loci than just 3 (or arguably 2)? What does this mean? Is this not a very genetic trait, or are there issues with measurement?
We thank the reviewer for this observation. We too expected that the use of dozens of thousands of participants would result in the identification of additional genomic regions.

However, our study, as well as the recently published paper following a similar premise using UKBB imaging data set (https://www.ahajournals.org/doi/10.1161/CIRCULATIONAHA.121.057709), identified only a few associated loci. Then, we can speculate that Df is a trait with reduced additive genetic variance compared to other retinal non-vascular traits, and its study with tens of thousands of participants elucidates few associated loci with a reduced effect size compared to the effect size of retinal non-vascular traits' SNPs.

Furthermore, we consider that the moderate statistical power in our GWAS is not influenced by the algorithm that calculates Df. A published study on Df following a similar protocol, above mentioned, calculated this trait from UKBB fundus images implementing a different algorithm and observed comparable findings. This remark is not only applicable to Df but also to supplementary GWAS of different retinal vascular traits (https://www.medrxiv.org/content/10.1101/2022.05.26.22275626v2.full).

6. The most convincing locus at OCA2/HERC2 is strongly associated with pigmentation. Might this still represent an artefact - might the image analysis software measure Df systematically differently in eyes of different pigmentation (which is not completely accounted for by simply adjusting for hair/skin colour)?
We would like to thank the reviewer for the feedback. We observed that Df has its strongest associations in genomic regions widely acknowledged in pigmentation studies. We then estimated a polygenic risk score for eye colour in the study population, as eye colour was not measured in UKBB (see line 525 in Methods section for further details). We included the eye colour PRS as a covariate in an additional GWAS model, which showed SNP effect sizes and P-values distributions equivalent to those obtained in our previous model without the PRS (see figure below). Furthermore, there is published GWAS on retinal phenotypes using UKBB which completed an additional GWAS model adjusting for average pixel intensity from the RGB image to evaluate the effect of image pigmentation. The results of this image-adjusted GWAS had similar effect and P values to those from previous GWAS that did not include the pigmentation adjustment. Finally, the effect of these loci on retinal vascular morphology have been described in our Df study, as well as in the previously published Df GWAS (https://www.ahajournals.org/doi/10.1161/CIRCULATIONAHA.121.057709) and published GWASs that were focused on different retinal traits and used non-UKBB datasets, suggesting that these genomic regions have an effect in multiple retinal traits (https://www.ncbi.nlm.nih.gov/pmc/articles/PMC4758888/, and https://pubmed.ncbi.nlm.nih.gov/21060863/).

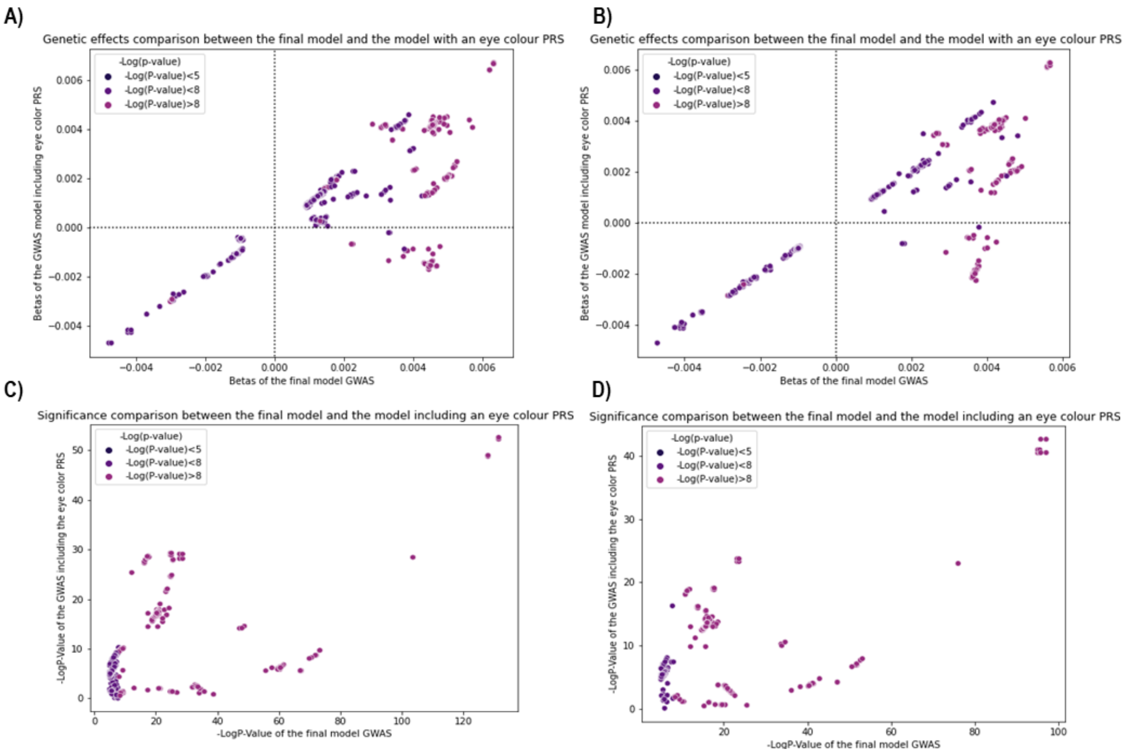

7. It is well-established that novel GWAS loci require replication in an independent cohort to have credibility. Given the availability of suitable cohorts (most notably the Canadian Longitudinal Study on Aging), I would suggest the authors seek to independently replicate their GWAS findings, as well as their prediction model findings.

We are unable to replicate the observations in an independent GWAS due to the lack of publicly available data. Our initial approach to address the replication matter was to perform independent Df GWAS with those UKBB participants with white European ancestry. We found that this complementary Df UKBB GWAS replicated the effect of associated SNPs in our main Df study. Furthermore, we contacted researchers using CLSA cohort for retinal analyses and they kindly agreed to estimate a polygenic risk score with our UKBB summary statistics for us. They next compared the estimated PRS with the phenotypic measures of their population. They did not share unpublished CLSA data from their participants and returned the phenotypic association between the PRS and Df and its significance. They analysed left Df, right Df and mean Df. This evaluation (see table below) showed that there is a direct and significant association between Df and the PRS. In fact, the effect size of the PRS in the CLSA association analysis is similar to the effect size obtained in the same assessment with the UKBB participants with a white European ancestry (see table below). Then, we found evidence that genetic determinants observed in UKBB are associated with the phenotypic measures of Df from a different cohort.

CLSA Models	Estimate	SE	P-value
Df (both eyes)* (n=16,205)	0.0212	0.0024	< 2E16
Df (right eye) (n=14,820)	0.0225	0.0026	< 2E-16
Df (left eye) (n=11,826)	0.0220	0.0030	5.33E-13

UKB Models	Estimate	SE	P-value
Df (both eyes)* (n=2,733)	0.0218	0.0079	7.65E-11
Df (right eye) (n=3,575)	0.0201	0.00874	1.11E-08
Df (left eye) (n=3,446)	0.0274	0.0083	4.4E-12

*measure from the best quality image of either sides, or average of both sides when both images were of similar quality

The replication of our genetic findings has been included in the results section of this manuscript from line 202 as:

"We complemented the replication of our GWAS results with an association study in the Canadian Longitudinal Study on Aging (CLSA). This consisted on fitting a linear regression on D_f that controlled for the 20 first principal components and a genetic risk score (GRS) for D_f, which was estimated using the summary statistics of the GWAS reported above (see methods). We found that the D_f GRS had a significant effect on left, right and mean D_f phenotypes (see Figure 3 e), suggesting thus that the SNPs previously described in the UKBB GWAS contribute to D_f variation in a different population".

Regarding predictive model replicability, our collaborators are completing an epidemiological study focused on Df and its relation with heart diseases; therefore they cannot share with us epidemiological data. The CLSA cohort has strict regulations regarding the use of the data in project proposals. The collaborators who completed the comparison with CLSA kindly modified they current project proposal in CLSA to include these additional analyses. An alternative to address the replication of our predictive model is to submit an additional project proposal and replicate our study with raw CLSA data, which would take one year approximately (e.g. considering the project approval and the completion of the replication) and would compete with the interests of our collaborators. Nonetheless, CLSA only contains 3-year follow-up data for its participants, it is expected then that there is not enough power and follow-up data to replicate the predictive model yet.

8. In addition to genetic correlation, might Mendelian randomisation approaches be helpful too? At places in the manuscript, the authors do start referring to one parameter causing the other, yet a genetic correlation does not suggest causality, and certainly not direction.

We would like to thank the reviewer for the feedback in this section. The promising genetic correlation estimates encouraged us to test causality with a Mendelian randomisation analysis (MR). We hypothesized that the genetic development of coronary artery disease, myocardial infarction, and stroke as well as a related medical condition, like hypertension or atherosclerosis might cause the Df variation. We then determined that each cardiovascular condition was an independent exposure and Df was the outcome. Sensitivity analysis for each independent assessment evidenced the presence of significant horizontal pleiotropy in the genetic instruments included for the MR, indicating that we are not able to infer the causality between such events.

Furthermore, we have reviewed the manuscript and edited those parts that might suggest the causality between common genetic associations in the retinal and global vascular health. As an example:

“All these estimates agree in direction with phenotypic correlations (see Supplementary Table 1) and published studies, which reported that retinal D_f decreases as people develop these conditions[2,3,11,42]. Therefore, our results suggest that these correlations of phenotypes could be partly explained by its shared genetic basis.”

9. I believe the authors have compared SCORE (age, sex, SBP and BMI) with 3 models incorporating either Df and/or the PRS. But the latter models also include smoking and there is no assessment of a model with SCORE + smoking. Clearly, smoking significantly affects retinal Df and significantly improves cardiovascular risk prediction tools (doi.org/10.1001/jamacardio.2021.4990). The authors should present a model of SCORE + smoking alone to make a true comparison of the added value of Df or PRS. The authors argue that the incremental benefit in discrimination between SCORE (age, sex, SBP and BMI) and a “random forest including Df” (age, sex, SBP, BMI, smoking and Df) is down to Df but smoking is in there too

The SCORE replica we developed did include smoking status and the omission of this fact in the submitted draft was a typo. We have modified the manuscript according to this missing information in both Results and Materials and Methods sections. To illustrate it, we included the following in line 247 within the Results section:

“Briefly, the model includes PRS_{CAD} derived from a meta-analysis completed by the CARDIoGRAMplusC4D Consortium[21], clinical variables from an established CAD risk assessment strategy named SCORE[22] (age, sex, smoking status, SBP and BMI), and the D_f of both eyes.”.

In Materials and Methods, we included the following in line 629:

“Our MI predictive model uses age at baseline, sex, systolic blood pressure, smoking status, BMI, and a polygenic risk score for CAD and D_f of both eyes separately as features in a classification algorithm.”

10. Prediction model reporting should adhere to TRIPOD guidelines. Many of the important items for reporting are missing in the manuscript. For example, how were missing data handled (e.g. missing covariables such as BP). Calibration as well as discrimination performance need to be presented.

We have adapted the manuscript to adhere to TRIPOD guidelines. As such, we detailed the process of data extraction, the presence of missing data in the population of study, and the lack of external validation. In particular, for the purpose of the first two missing items, we included:

“We then extracted the information of the established risk variables, that is, age, sex, SBP, BMI and smoking status, for the population of study using the curated phenotypes from UK Biobank July 2017 release30. The population of study for the predictive models did not contain missing data. In fact, we extracted controls only considering those UKBB participants with no missing data and both Df measures, as the majority of individuals with a Df measure were healthy. 56 MI cases had a missing Df measure from one eye. In these cases, we did not predict the missing value and only used the available Df measure**.”**

Regarding the lack of external validation, we described: “Fourth, we did not have an external validation cohort to complete an external validation of our MI model. This matter is attributed to the lack of available datasets containing extensive phenotyping from its participants”.

11. The Kaplan-Meier survival analysis is unclear. Did the authors censor participants who died from a non-MI event? It is unclear why the KM curves drop only at yearly intervals when

the linked outcome data provide more precise timings.
We would like to thank the reviewer for the comment. The Kaplan-Meier was generated with incident MI events and we did not censor participants that died from non-MI events. This lack of censoring was attributed to the fact that most deaths (n=35) took place after the studied timeline and that there was a minority of cases (n=6) that died soon after the MI event. The MI events and the MI date data in the population of study derived from secondary care and self-reported data. The Kaplan-Meier plot from the manuscript was created using finite years as unit for the x-axis and therefore, the curve drops at the end of each year within the timeline. We have modified the plot and changed the units of the x-axis to days so it displays a more precise timing on the MI events and the difference between populations, see the following plot.

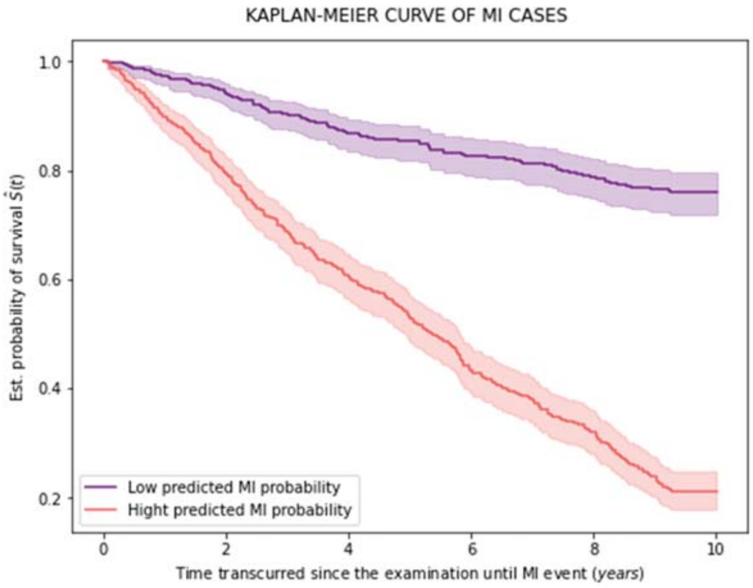

Minor corrections:

12. Is the term "fine-mapping" used in the title reasonable? Perhaps there are different definitions, but this seemed like a straightforward GWAS to me without any fine-mapping of causal variants at each locus.
We have modified the GWAS results section by describing the fine-mapping results at the beginning of this section. This adjustment was completed to highlight that associated lead SNPs derive from credible SNPs sets (PIP>0.95) obtained through fine-mapping analyses using SUSIE.

13. Why was there no cross-ancestry GWAS to maximise power?
We thank the reviewer for this comment. We completed our fractal dimension GWAS in ancestry-specific populations due to the lack of publicly available data to replicate the GWAS results and for completion of a fine-mapping analysis. We considered that the largest ancestry population (white British) in UKBB would be used for our main analysis and white European, African, and Asian ancestries GWASs could be used for replication. We used non-white British ancestries to illustrate that the associated Df loci had a comparable effect across ancestries. Furthermore, fine-mapping analysis should be completed using one ancestry only as it thoroughly investigates the effect of the genomic regions on the explored trait.

14. Why was the Wilcoxon test used to compare models? These are nested models comparing SCORE to the addition of Df (and smoking). Why not just use a chi-squared likelihood ratio test, which is considered the gold standard by prediction model experts (see Frank Harrell Jr's work on this)?
We would like to thank the reviewer for this comment. We used a Wilcoxon test to compare the AUC estimates between two models that were validated using the same data partitions of non-normally distributed features. Our work has the limitation that it cannot apply a chi-squared likelihood ratio test between the presented models and the baseline, as it is only applicable to regression models. The model we presented here is based on a random forest classifier and the importance score of the independent variables does not reflect a linear/logistic association with the outcome.

15. Please provide a clear rationale for comparing with SCORE rather than other prediction models. SCORE2 is developed and mostly used in European countries, QRISK3 is used in the UK. Both are far better than SCORE.

We selected SCORE classifier as it is the baseline model implemented in previous efforts that leveraged retinal vascular information (https://www.nature.com/articles/s41551-018-0195-0 and https://academic.oup.com/cardiovascres/article/117/8/1949/5880582?login=true#267330466). Additionally, the risk factors included in SCORE are very similar to QRISK3 and SCORE2, albeit SCORE does not take into account population-specific effect in risk factors. However, in our work the population characteristics are similar among cases and controls as all derive from a white British or white European non-British populations that reside in the UK.

16. Please provide more detail and explanation for the matching. While the matching was only on age, there remains a reasonable difference in age post-matching (54.2 versus 57.3). However, there is no sex matching despite retinal Df and MI incidence differing in these groups (the same with hypertension and diabetes).
Controls in the population of study for the predictive model were age matched. In particular, we established that the controls' age range was equivalent to those from the cases (i.e. 45-61 years). We then randomly chose an equal number of participants with good-quality images of both eyes, no cardiovascular event within the CAD spectrum, and no known risk factor (e.g., diabetes, hypertension, and family history of heart disease). We observed that there is a statistical age difference between both populations, which could be influenced by the total numbers of cases and controls, but even so, this variables were included in the model to account for its effect.

We further investigated the influence of the significant age difference by creating an additional control set that was age and sex matched. In this case, we selected a sex-matched control for every case that had equal age. This constraint led to a dataset unbalance given that there were cases with no age and sex matched controls. This dataset contained 526 cases and 397 controls. We trained our proposed model using this novel dataset. Internal 10FCV indicated that our model outperformed SCORE with greater performance metrics (see Table below). Nonetheless, precision, specificity, sensitivity and AUC values were smaller than those obtained with our previous dataset. This reduction can be attributed to the latest case-controls match, which created an unbalance in the population causing a statistical power loss . Thus, we found evidence that our former population of study achieved a better statistical power to identify cases and controls, even if controls were matched through age ranges.

Furthermore, our study population for the predictive model did not include individuals with diabetes. This was because diabetics have comorbidities directly affecting theYe retinal vasculature. This rationale agrees with a recently published study

(https://pubmed.ncbi.nlm.nih.gov/35852586/), that indicates that diabetics and the general population present different associations with cardiovascular events. As for hypertension, controls were selected to not have risk or family risk of these conditions.

Model	MI			
	Precision (95%CI)	Sensitivity (95%CI)	Specificity (95%CI)	AUC (95%CI)
SCORE model[16]	0.612 (0.584-0.649)	0.777 (0.731-0.796)	0.631 (0.603-0.672)	0.596 (0.561-0.633)
Random Forest including PRS_{CAD}	0.701 (0.668-0.739)	0.775 (0.732-0.803)	0.683 (0.647-0.714)	0.667 (0.635-0.698)*
Random Forest including mean Df and PRS_{CAD}	0.662 (0.623-0.697)	0.749 (0.708-0.783)	0.679 (0.641-0.703)	0.649 (0.612- 0.676)*
Random Forest including Df and PRS_{CAD}	0.705 (0.671-0.736)	0.783 (0.759-0.818)	0.710 (0.684-0.746)	0.678 (0.639-0.712)*

17. Please consider swapping precision and recall for sensitivity and specificity. The matched cohorts are balanced in numbers so the benefit of PR is limited and this metric is more familiar to computer science/information retrieval/engineering audiences. Also, given these are cardiovascular risk prediction models, comparison to other literature will mainly be through sensitivity/specificity.

The performance evaluation of our presented models as well the baseline strategy now contains precision, sensitivity, specificity and AUC (see table below). We have maintained the precision measures from the models to facilitate its interpretation in multiple disciplines, because, as mentioned by the reviewer, precision is commonly implemented data science strategies, which might also investigate the potential of retinal information. The observations of these estimates are equivalent to those described in the previous manuscript, as our proposed models yielded a higher AUC, precision, sensitivity, and specificity than those obtained from SCORE. The new version of the manuscript includes the table below as Table 3.

	MI			
Model	Precision (95%CI)	Sensitivity (95%CI)	Specificity (95%CI)	AUC (95%CI)
SCORE model[16]	0.716 (0.664-0.741)	0.725 (0.691-0.767)	0.691 (0.652-0.731)	0.719 (0.681-0.737)
Random Forest including PRS_{CAD}	0.735 (0.708-0.782)	0.756 (0.726-0.801)	0.739 (0.702-0.777)	0.741 (0.725-0.775)*
Random Forest including both eye-specific Df	0.756 (0.732- 0.802)	0.778 (0.762-0.831)	0.758 (0.721-0.795)	0.763 (0.750-0.802)*
Random Forest including mean Df and PRS_{CAD}	0.733 (0.716-0.770)	0.779 (0.743-0.814)	0.756 (0.717-0.797)	0.748 (0.722- 0.773)*
Random Forest including Df and PRS_{CAD}	0.770 (0.734-0.805)	0.790 (0.757-0.826)	0.764 (0.728-0.800)	0.770 (0.751-0.802)*

18. Please can it be clarified whether the Df value in the risk prediction model is in some way adjusted by image quality? Apologies if this is somewhere in the methods but I could not find it, perhaps it can just be mentioned in the Results section near line 265. Yes, we trained an additional model that included Df adjusted by image quality score. The performance of this model is reported in the table below, included in the manuscript as Supplementary Table 10. Results are consistent with those from our initial approach that did not consider the effect of IQS. We have modified the Results section to clarify this analysis, see lines 276, as follows:

"Additional assessments in our proposed model indicated that the replacement of D_f measures with D_f adjusted by IQS, the introduction of one-eye D_f measurements in our MI model, or the use of mean D_f in our model yielded a comparable performance to the aforementioned ones."

	MI		
Model	Precision	Recall	AUC
Random Forest including left Df	0.756±0.001	0.773±0.002	0.761±0.001
Random Forest including left Df and PRS_{CAD}	0.759±0.001	0.793±0.001	0.774±0.001
Random Forest including right Df	0.759±0.001	0.778±0.002	0.765±0.001
Random Forest including right Df and PRS_{CAD}	0.756±0.001	0.787±0.001	0.772±0.001
Random Forest including quality-adjusted Df	0.766±0.001	0.784±0.002	0.771±0.001
Random Forest including quality-adjusted Df and PRS_{CAD}	0.768±0.001	0.796±0.001	0.774±0.001

19. Rather than SE, confidence intervals would be more informative in Table 3 and when presenting performance metrics on discrimination. It is easier to see overlap between models.
The resubmitted version of the manuscript includes the CI for all the performance metrics for the predictive models. As shown in the Table inserted in question 17, the CI for the AUC across the validated models indicates that our proposed models outperform the MI risk classification than SCORE. Furthermore, it can be observed that the model including both eyes Df and the PRS achieves a similar performance to the random forest classifier (RFC) including both eyes RFC and not including the PRS. Thus, these observations are consistent with those from the paired comparisons using Wilcoxon ranked test across all evaluated models.

20. Please temper down any generalisability claims of the prediction model for MI. This was developed on a particular population with a small dataset who have a narrow sociodemographic profile.
We have modified the manuscript accordingly to the reviewers' observation. Additionally, we expounded our limitations in the discussion, pointing out that our results apply to a particular sample of UKBB and further studies and external validation are required to corroborate its generalizability. This matter is now detailed in line 426 as follows:

"Secondly, the study population for the predictive models only consisted of UKBB participants with European ancestry with similar sociodemographic status, restricting the application of translational strategies to non-white European and British individuals among different sociodemographic profiles. Furthermore, the PRS included in the proposed predictive model is based on a meta-analysis completed with participants with mainly white European non-British and white British ancestries. Then, it is of utmost importance to complete GWAS in non-European populations to provide input for PRS estimations so that they are included in such medical applications."

21. Some of the terminology is a little loose - e.g. lines 89-90: the loci are at or near the genes (i.e. better to say the loci "at" or "near" SLC12A9 and RDH5, as the loci do not cover a whole gene and may not even be functionally related to the gene).

The latest version of the manuscript has implemented this observation in the results and discussion section.

22. Line 169: references missing

We have included suitable references in the manuscript (see line 178) as follows: "Recent ocular studies demonstrated their implication in lens disorders, cataract, glaucoma, visual acuity, and retinal venular and arteriolar width and tortuosity[5,17,20,28,32–40])."

23. Line 496: how were British individuals identified? How were unrelated participants selected?
We established the ancestry and the relatedness of our population of study using the ancestral classification and the kinship coefficients generated by Canela-Xandri et al. (https://www.nature.com/articles/s41588-018-0248-z) and UKBB. In our study, we selected that unrelated participants should have a kinship coefficient <0.0442. This information has been included in line 516 of the manuscript to clarify the quality control of the study population as: "Unrelated individuals were selected using a 0.0442 threshold from UKBB and a previous work that established unrelated UKBB participants with a white British ancestry[30]"

24. Line 579: please explain the P-value threshold choice of 0.001
We selected a 0.001 threshold for the Protein-Protein Interactive Network analysis to consider those networks that had a significant association with the associated genes and transcription

factors. The decision of a more restrictive threshold was motivated by the fact that these genes might influence multiple pathways that affect general biological processes.

25. Line 595: could you please elaborate on “the approximate period when participants underwent the ophthalmic examination”.

Most participants underwent their ophthalmic examination as part of their baseline UKBB assessment. Nonetheless, there are a couple hundred participants that had to attend to a different centre to complete the ophthalmic examination, as only 6 UKBB centres completed this additional evaluation. In these cases, there is no information about the date of the event and as such, we assumed that this additional assessment was completed at a close time from their general UKB assessment.

REVIEWER #2 (REMARKS TO THE AUTHOR):

MAJOR CORRECTIONS:

1. To clarify, the GWAS on Df was performed on 38,811 and 38,017 participants that had right and left Df measurements, were there overlapping people in this or were these 2 completely separate groups?

In the genetic analysis of this work, left and right Df populations had a majority of overlapping participants (n=37,712) from the UKBB. Nonetheless, there were some individuals that had a Df measure from just one eye that are included in only one GWAS analysis, caused by quality differences between left and right images or due to missing fundus image from one of the eyes.

2. How significant (from a clinical stand point) is an increase in the AUC from 0.741 (SCORE) to 0.77? The ability to stratify patients by MI risk was very compelling.

We evaluated the statistical difference between AUC parameters from the tested models by implementing a Wilcoxon rank test. As shown in the Table below, these indicated that there is a significant difference between AUC estimations from SCORE and our proposed model and its variations. Additionally, now we have computed the 95% CI estimates for the performance metric to facilitate the comparison between models (see response to reviewer 1, question 17). The observations from the 95% CI agree with the Wilcoxon tests that our model and its variations yield a significantly greater AUC, recall, precision, and specificity. Furthermore, our model outperformed the case-control discrimination completed by SCORE, as we found that our MI model correctly identified 32 cases and 131 controls which were falsely labelled in SCORE predictions. Therefore, our results indicate that fractal dimension provides additional predictive value to identify high-risk MI cases in this population of study. We also need to point out that one limitation of this study is that we are unable to externally validate the performance of our proposed model, which is required for its implementation in clinical settings.

Models comparison	Wilcoxon signed rank sum test	P-Value*
SCORE VS RFC with D_f and PRS_{CAD}	1.0	0.0039
SCORE VS RFC with D_f	1.0	0.0039
SCORE VS RFC with PRS_{CAD}	6.0	0.027
RFC with D_f and PRS_{CAD} VS RFC with PRS_{CAD}	7.0	0.037
RFC with D_f and PRS_{CAD} VS RFC with D_f	25.0	0.846
RFC with PRS_{CAD} VS RFC with D_f	7.0	0.037

3. Linking Df to MI via Notch and VEGF signaling is interesting. I did find the discussion about this to be confusing, are you suggesting that Notch signaling is being disrupted, leading to inhibition of Notch and VEGF signaling, which would lead to worse outcomes, or that the disruption is beneficial? Some more clarification around the discussion on this would be helpful.

We would like to thank the reviewer for this comment. We found that the allelic substitution in Df-associated regions predicted a disruptive transcription activity of 5 TFBS and 9 associated genes that participate in Notch-VEGF signalling. Additionally, the literature indicates that the activity of these transcription factors and genetic variation upregulate of Notch signalling pathway and leads to better short and long-term outcome. From our analysis, we observed that reference alleles in two TFBS (ESRRA and HES1) have a positive effect on Df. Considering these facts together, we speculate that individuals with higher Df might not have a disrupted notch signalling pathway that might be protective towards the response of a myocardial infarction. Notwithstanding this, further animal models and in-vitro studies at different physiological and pathological stages of the retina and MI are required to further investigate and consolidate our hypothesis. We have modified the discussion of the paper to clarify this point in line 355, as follows:

“We complemented our functional analysis with in-silico TFBS disruptiveness prediction of credible variants. We observed four credible D_f gene sets with a strong disruptive effect in 5 TFBS and 9 regulated genes, which participate at different Notch signalling pathway stages (Fig. 6). One possible mechanism to modulate its activity is through the alteration of *ESRRA* binding affinity, which influences *VEGFA* transcription. In-vitro and animal model studies indicate that after an MI event, *VEGFA* upregulation activates *VEGF* signalling pathway, which has a crosslink with Notch pathway and increases its activity[43,52–54]. Another mechanism derives from *HES1* binding site affinity. *HES1* influences *MAML1* and *NOTCH1* expression and directly affect Notch signalling[43–45,55]. The last mechanism influencing Notch activity is mediated through *TBX20* binding site affinity, which plays a role in *TLE3* transcription. Under a MI event, multiple studies indicate that *TLE3* upregulation activates PI3K/Akt signalling pathway, a downstream process of Notch signalling pathway[56,57]. Numerous in-vitro and

animal models studies support that this increased Notch activity, mediated by *HES1*, *ESRRA*, and *TBX20* upregulation, leads to reduction of cellular oxidative stress consequently improving myocardial viability, regeneration, and survival rate after a MI event[43–45]. We hypothesize that the TF binding disruption caused by these genetic variants influence Notch activity and, in the case of MI, might have a risk conferring effect[44,45,55–61]. Furthermore, the alleles which predict a basal TFBS activity have a positive effect size on D_f, so we could speculate that these individuals might not have a disrupted Notch signalling pathway, which might be protective towards the response of a myocardial infarction event. An extended discussion is available in the Supplementary Materials. Thus, these analyses suggest that there is an intricate shared genetic basis between vascular complexity and MI and further *in-vitro* experiments are needed to characterise gene expression and regulation of retinal tissue to better understand it.”

MINOR CORRECTIONS:

1. Line 171, should read Fig 3d rather then 2d

We have modified the manuscript version with the adequate figures referencing (see line 180).

2. In the extended discussion you have "W hypothesize" instead of "We"

We have corrected the spelling of this word in the extended discussion.

REVIEWERS' COMMENTS:

Reviewer #1 (Remarks to the Author):

The authors have thoughtfully responded to all comments, and have mostly addressed my concerns.

Reviewer #2 (Remarks to the Author):

I am happy with the responses to all reviewers comments, thank you
